# Genetic Optimization Algorithm for Metabolic Engineering Revisited

**DOI:** 10.3390/metabo8020033

**Published:** 2018-05-16

**Authors:** Tobias B. Alter, Lars M. Blank, Birgitta E. Ebert

**Affiliations:** Institute of Applied Microbiology—iAMB, Aachen Biology and Biotechnology—ABBt, RWTH Aachen University, Worringerweg 1, 52074 Aachen, Germany; tobias.alter@rwth-aachen.de (T.B.A.); lars.blank@rwth-aachen.de (L.M.B.)

**Keywords:** metabolic strain design, heuristic optimization, constraint-based modeling

## Abstract

To date, several independent methods and algorithms exist for exploiting constraint-based stoichiometric models to find metabolic engineering strategies that optimize microbial production performance. Optimization procedures based on metaheuristics facilitate a straightforward adaption and expansion of engineering objectives, as well as fitness functions, while being particularly suited for solving problems of high complexity. With the increasing interest in multi-scale models and a need for solving advanced engineering problems, we strive to advance genetic algorithms, which stand out due to their intuitive optimization principles and the proven usefulness in this field of research. A drawback of genetic algorithms is that premature convergence to sub-optimal solutions easily occurs if the optimization parameters are not adapted to the specific problem. Here, we conducted comprehensive parameter sensitivity analyses to study their impact on finding optimal strain designs. We further demonstrate the capability of genetic algorithms to simultaneously handle (i) multiple, non-linear engineering objectives; (ii) the identification of gene target-sets according to logical gene-protein-reaction associations; (iii) minimization of the number of network perturbations; and (iv) the insertion of non-native reactions, while employing genome-scale metabolic models. This framework adds a level of sophistication in terms of strain design robustness, which is exemplarily tested on succinate overproduction in *Escherichia coli*.

## 1. Introduction

Metabolic engineering aims to enable the production of pharmaceuticals, fine chemicals, and fuels by microbial cell factories, and strives to maximize productivity and profits [1]. In the last 30 years, advances in DNA sequencing and systems analytical technologies have led to an immense expansion of integrated knowledge about genetics, biochemical metabolic pathways, and their regulation, and enabled researchers to specifically understand and manipulate microbial metabolism [2].

From the sheer metabolic and regulatory network complexity a key problem of metabolic engineering approaches falls into place: How does one intervene in those biochemical networks to reach or approach an engineering aim with a reasonable investment of time, money, and materials? The use of computational models of metabolism seeks to answer these and related questions by facilitating the integration of biochemical knowledge and *omics* data. Techniques, such as flux balance analysis [3], elementary modes analysis [4], or flux variability analysis [5], helps to explain metabolic properties and to predict the effect of genetic perturbations on microbial metabolism. By incorporating routines, which systematically search for intervention sets that yield a desired phenotype (e.g., a target product yield), a panoply of variants of these computational methods has emerged [6] to support metabolic engineers to most effectively interpret the information content of metabolic models.

The search for an optimal genetic intervention set typically poses a nested, bilevel-optimization problem. The outer problem optimizes an engineering objective by varying the network structure through gene knockouts, knockdowns, or overexpressions. The inner problem returns the microbial phenotype for a given intervention strategy based on a cellular objective, from which the outer objective function is evaluated. By exploiting basic theorems of linear algebra, such bilevel problems are usually transformed into single-level mixed-integer linear or quadratic programming problems and solved using powerful mathematical programming algorithms [7,8,9,10,11]. The usefulness of these frameworks in aiding metabolic engineering projects has been demonstrated for various microbial strains and target compounds [12,13,14,15,16], but still, practical applications lag behind the vast efforts put into theoretical studies.

Solving bilevel optimization problems using gradient search techniques bears two major drawbacks. Firstly, the necessary mathematical transformations increase the complexity of the problem formulation, thus limiting the tractable number of simultaneous interventions per simulation. More importantly, only linear constraints and objective functions can be used in these frameworks, which may not be appropriate for representing biological objectives [6].

The application of metaheuristics as search routines circumvents the formulation and complexity problems of the classical gradient search techniques. Metaheuristics is a general term for optimization techniques, which are to some degree based on random variables to derive optimal solutions to an optimization problem [17]. Evolutionary or genetic programming is one prominent example among metaheuristic methods, which adopts the principles of biological evolution for finding (near-)optimal solutions to optimization problems and is particularly advantageous when dealing with complex, high-dimensional objective functions and constraints [18]. The genetic algorithm (GA) evolves an optimal genetic intervention set for a given metabolic engineering objective by a systematic and repeated selection, crossover, and mutation of a population of solutions [19,20,21]. Such a search heuristic allows for an efficient integration of any complex prediction method for microbial mutant phenotypes, such as Minimization of Metabolic Adjustment (MOMA) [22], as well as the consideration of sophisticated, non-linear engineering objectives as fitness functions. By applying, e.g., OptGene, theoretical studies [23,24,25], but also practical implementations of identified genetic intervention strategies [26,27,28,29] have proven the benefits of GAs for the identification of overproduction strain designs.

A variety of metaheuristics as optimization algorithms have already been applied for the computation of metabolic engineering strategies [30,31,32,33]. We chose to apply GA for microbial strain design purposes because of its intuitive optimization principles and already-proven usefulness in this field of research. Even though the versatility and flexibility of the GA framework has previously been emphasized [19,25], this appealing feature was never exhausted in terms of the integration of various established engineering objectives and approaches, aiming to yield robust production strain designs. Additionally, with regard to future considerations of models, constraints, and engineering, as well as biological objectives of growing complexity, we sought to shed light on the behavior and performance capabilities of GAs.

Due to the nontransferable behaviors of GAs among different optimization problem classes [34], we first explored the dependencies between the optimization parameters of GAs and classical model-based metabolic engineering problems. To this end, we conducted sensitivity analyses for the mutation rate, population size, number of generations, etc., while focusing on the ability to converge to optimal strain design solutions for overproduction of target molecules in *Escherichia coli*. We particularly examined the importance of the duality between diversification and intensification (also known as exploration and exploitation) of solution candidates for circumventing premature convergence. Secondly, we demonstrated, and eventually expanded, the GA’s versatility. We included the simultaneous evaluation of multiple cellular objective functions to derive pareto-optimal, robust strain designs. Inspired by the OptStrain framework [8], we additionally implemented a routine to insert novel network edges taken from a preprocessed pool of candidate reactions at runtime. Moreover, a strategy was derived and implemented to simultaneously minimize the number of interventions (e.g., gene deletions) while optimizing for the chosen engineering objective.

In summary, we investigated and exhausted the behavior and performance capabilities of GAs for metabolic engineering approaches and, beyond that, integrated previously-independent design objectives and methods in one framework. Hence, we promote the use of GAs for sophisticated metabolic models demanding high computing power [35], as well as the need to solve engineering problems of growing complexities.

## 2. Materials and Methods

### 2.1. A Basic Genetic Algorithm for Metabolic Engineering

The GA is a randomized but directed search and optimization method modeled by the principles of natural selection. It iteratively evolves a set or population of solutions to an optimization problem (a solution will be referred to as an individual), i.e., towards better solutions, while eventually converging at optimality. According to Srinivas and Patnaik [36], the key characteristics of a GA are:

1. A genetic representation of solutions. Here, we employ a binary coding.
2. Populations of individuals as evolutionary communities.
3. A fitness function for evaluating the goodness of individuals.
4. Operators, which generate a new population from an existing one and which can be controlled by parameters that shape the fitness-related or random transformation behavior.

**Figure 1:**
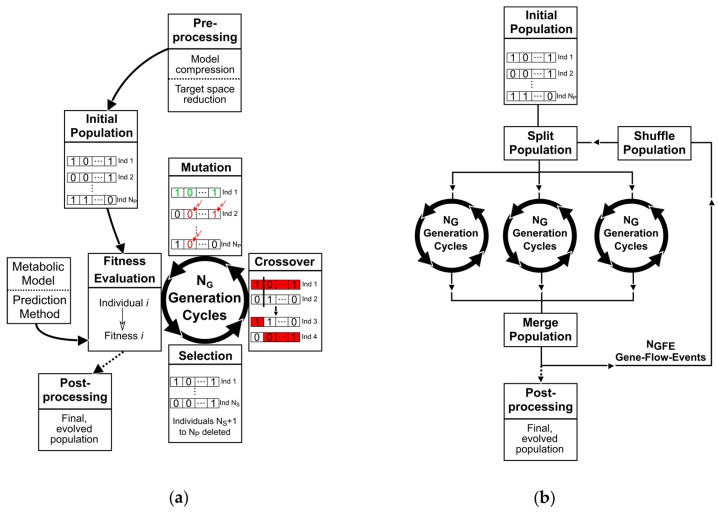
(**a**) Scheme of a basic GA; and (**b**) illustration of the parallelization method.

These characteristics have already been shown to be advantageous for in silico metabolic engineering approaches in finding a set of reaction knockouts, which optimizes overproduction [19,21,25]. Therefore, we implemented a GA using the basic structure of the OptGene framework [19] as well as the descriptions of Haupt and Haupt [37] as a starting point. The principle scheme of the GA is sketched in Figure 1 including the selection, mating, mutation, and fitness evaluation operator constituting the core GA, as well as a pre- and post-processing routine. Successive application of each GA operator to a population will be called a generation in the following. In a nutshell, GAs iteratively create new and eventually better knockout strategies from an evaluated set or population of solutions by (i) selecting the best solutions (selection); (ii) pairwise substituting parts of the knockout strategies (crossover); and (iii) randomly changing reaction or gene deletion targets of the new solutions (mutation). The key parameter and technical terms are described in the following sections.

#### 2.1.1. Population of Binary Individuals

In terms of a strain design problem, an individual represents a set of reaction or gene deletions. Following characteristic (1), each of the $N_{D}$ deletions of an individual (refer to the list of symbols at the end of the text), in the following termed as a (gene or reaction) target, is encoded by a binary number consisting of a sequence of $N_{B}$ bits. Within the GA framework, each value of this binary number is assigned to a specific reaction or gene that is to be deleted if it occurs in an individual. In the GA developed in this work, the number of targets per individual $N_{D}$ is user-defined and fixed. However, the number of actual reaction deletions applied to a metabolic model may be smaller than $N_{D}$ if two or more targets within an individual point to the same network perturbation.

Now consider the problem of assigning $N_{T}$ possible reaction or gene targets within a network, which is defined as the target space of a metabolic engineering problem, to $2^{N_{B}}$ permutations of a binary number. For example, a target space consisting of 200 reactions can be encoded by a binary number with at least eight bits. In this case, $2^{8}=256$ values can be represented by eight bits, hence, just exceeding the number of reactions within the target space. However, assigning each target reaction to a value of the binary number leaves 56 permutations of the binary number unassigned. If these are left unassigned, the chance of encountering individuals encoding for less than $N_{D}$ deletions varies with the target space size. To avoid this bias towards the number of deletions per individual, we chose to also assign blank binary values to reactions or genes in the target space. To additionally guarantee comparable probabilities for each target to occur we increased the number of bits $N_{B}$ per binary number so that each target in the target space is assigned to at least 50 binary values. The number of bits were calculated using Equation (1), which was derived from considerations of how to choose a minimal $N_{B}$ sufficient to encode $50\cdot N_{T}$ values:

(1) $$N_{B}=\mathrm{Round}\left(\frac{\log\left(50\cdot N_{T}\right)}{\log\left(2\right)}\right).$$

Consequently, the maximal difference in the probability of drawing two independent targets is less than 2%. Using this specific binary representation, a maximal, user-defined number of targets per individual is guaranteed.

At the start of the GA, a population of $N_{P}$ individuals, each consisting of $N_{D}$ binary numbers of size $N_{B}$, is initialized. The initial state of each binary number within the population’s individuals is selected randomly. As mentioned in characteristic (2), a population is an evolutionary community meaning that all the GA operators described in Section 2.1.3 and Section 2.1.4 act on populations as a whole to approach optimal solutions.

#### 2.1.2. The Fitness Function

The fitness or goodness F of individuals quantifies to which extent metabolic network perturbations facilitate overproduction of a target molecule or, in general, comply with the engineering objective. As an engineering objective, we chose the biomass-product coupled yield (BPCY) [19,23], which is calculated by:

(2) $$BPCY=\frac{v_{P}\cdot \mu }{v_{S}},$$

where μ depicts the growth rate, and $v_{P}$ and $v_{S}$ are the product formation and substrate uptake rates, respectively. The three parameters in Equation (2) are taken from phenotype predictions of microbial mutant strains. These phenotype predictions, namely, the mutant flux distributions, are calculated using the Minimization of Metabolite Balances (MiMBl) method [38], which may be considered as an adaption of the Minimization of Metabolic Adjustment (MOMA) algorithm [22]. MiMBl minimizes the distance between the mutant and a reference metabolite balance vector. Since this work solely focused on *E. coli*, we obtained our reference state from the experimental results of Ishii et al. [39]. For more detailed descriptions we refer to Supplementary I.1.

#### 2.1.3. Selection, Mating, and Crossover

As a first step in a generation, the best $N_{S}$ individuals are selected for mating according to their fitness, whereas all other $N_{P}-N_{S}$ of the $N_{P}$ individuals in the population are deleted. $N_{S}$ is calculated by:

(3) $$N_{S}=N_{P}\cdot X,$$

with X being the user-defined selection rate ranging between 0 and 1.

The mating pairs for crossover are assembled using a roulette wheel weighting approach. Therefore, two individuals are randomly selected from the pool of $N_{S}$ selected solutions to form one pair. The probability $P_{i}$ of selecting a specific individual is thereby proportional to its fitness value $F_{i}$ and is calculated according to Equation (4):

(4) $$P_{i}=\frac{F_{i}^{*}}{\sum_{i}^{N_{S}}F_{i}^{*}},$$

with:

(5) $$F_{i}^{*}=F_{i}-F_{R},$$

where $F_{i}$ is additionally normalized by the fitness $F_{R}$ of the best discarded individual. However, if one or more selected individuals exhibit zero fitness, a minimal probability $P_{min}>0$ is guaranteed by scaling $F_{i}$ according to Equation (6):

(6) $$F_{i}^{*}=F_{i}+\frac{P_{min}\cdot \sum_{i}^{N_{S}}F_{i}}{1-P_{min}\cdot N_{S}}$$

If not noted otherwise, $P_{min}=0.1\cdot N_{S}$.

Since during selection the worst individuals are deleted, these $N_{P}-N_{S}$ empty spaces in the population have to be filled by the offspring of the mating pairs to keep the original population size constant. Therefore, $N_{P}-N_{S}/2$ mating pairs are sampled because two offspring are generated by the crossover of two mated individuals. Avoidance of two identical mating pairs is set as a criterion for exclusion during sampling.

Crossover, the creation of offspring individuals from mating pairs, is initialized by a random selection of a crossover point or kinetochore for each mating pair. Kinetochores are restricted to positions in between two neighboring genes. The genes left of the kinetochore of the first parent are merged with those to the right of the kinetochore of the second parent to form the first offspring individual. The second offspring is created complementarily with the remaining parent genes (Figure 1a).

#### 2.1.4. Mutation and Elitism

The mutation operator drives the exploration of the target space by randomly changing deletion targets within individuals of a population with a given probability. One random number is drawn for every bit of an individual’s binary numbers encoding for the deletion targets. In case of a mutation event, the bit value is inverted. The probability with which a 1 is turned into a 0, or vice versa, is set by the user-defined mutation rate R, ranging between 0 and 1. This process eventually changes the value of a binary number such that the corresponding deletion target and the individual’s phenotype change.

We additionally adapted the concept of elitism and, hence, the best parent individual is not mutated. After mutation, a new generation is propagated by calculating the fitness of the novel individuals.

#### 2.1.5. Parallelism

To exploit multi-core and multi-threading processor architectures, several independent generation strands are processed in parallel (Figure 1b). Therefore, an initial population is randomly split into $N_{C}$ subpopulations of equal size. Each sub-population is passed to a separate thread and undergoes independent evolution following Section 2.1.3 and Section 2.1.4. After $N_{G}$ generations, the final subpopulations of the generation strands are merged into one population. We will refer to this as a gene-flow event (GFE) in accordance to classical genetics where “gene-flow” describes an exchange of genetic variation between two separated and independently evolved populations. Following a GFE, the process comprising the division of the complete population, distribution to the available threads and independent evolution of the subpopulations is repeated. After $N_{GFE}$ GFEs, the GA is terminated, resulting in a final population of $N_{P}$ evolved individuals.

### 2.2. Adaptive Probabilities of Mutation

Previously, auxiliary GA operations have been derived that adapt GA parameters at runtime to simultaneously maintain a sufficient level of diversity of individuals within a population and the convergence capacity of GAs [18,36]. To test and verify the described advantage of such techniques for metabolic engineering problems, we implemented a strategy to adapt the mutation rate for each individual following the work of Srinivas and Patnaik [36]. The mutation rate is made dependent on the relative fitness value of an individual as well as the diversity of its population and is calculated by:

(7) $$X_{i}=\frac{F_{max}-F_{i}}{F_{max}-F\prime }\left(X_{max}-X_{min}\right)+X_{min},$$

with $F_{max}$ being the fitness of the best individual within the population and F′, the population’s mean fitness. Equation (7) ensures that $X_{i}$ ranges between a pre-defined minimal and maximal mutation rate $X_{min}$ and $X_{max}$.

### 2.3. Additional Features

#### 2.3.1. Gene Deletion Targets

To make use of the complex gene-protein-reaction (GPR) associations inherent to many metabolic models, we enabled the possibility of computing gene rather than reaction deletion target-sets. Since any fitness function evaluation employing metabolic models demands the specification of reaction network perturbations, we implemented a routine, which translates simultaneous gene deletions to reaction deletions according to the logic operations given by the GPRs.

#### 2.3.2. Multi-Objective Optimization

To simultaneously optimize multiple engineering objective functions, the fitness function was expanded by the OptKnock [40] and gcOpt [41] methods. Both OptKnock and gcOpt are originally formulated as bilevel optimization problems. Optknock identifies strain designs that maximize the target production rate at maximal growth, whereas gcOpt determines knockout strategies leading to growth-coupling by maximizing the minimally guaranteed production rate at a medium growth rate. Consequently, we derived a multi-objective fitness function by integrating a linear combination of the production rate at maximal growth (OptKnock), the growth coupling strength (GCS, gcOpt) and, as explained in Section 2.1.2, the BPCY. Each objective function value is normalized by their maximum to ensure uniform value ranges between 0 and 1. Additional, independent weighting factors can be applied to each objective function, but were neglected in this work.

The GCS has originally been defined as the ratio between the mutant’s total yield space area below the upper yield space bound and the area below the lower yield space bound, which is inaccessible to the mutant [41]. Here, we simplified the calculation of the GCS to reduce the computational burden while guaranteeing a meaningful approximation of the GCS measure. For a detailed description of the original and simplified definition of the GCS refer to Supplementary I.2.

#### 2.3.3. Minimization of Perturbations

We incorporated a fitness transformation routine to facilitate the minimization of simultaneous genetic perturbations while evolving overproduction individuals. Our approach is to scale the fitness $F_{i}$ of an individual i (here referred to as the objective fitness), which stems from the evaluation of the cellular objective function, by the number of unique reaction or gene deletions $I_{i}$ of *i* according to Equation (8):

(8) $${\hat{F}}_{i}=F_{i}+F_{i}\left(I_{max}-I_{i}\right),$$

where ${\hat{F}}_{i}$ is the scaled fitness and $I_{max}$ denotes the maximal possible number of unique perturbations per individual.

By applying Equation (8), the fitness of individuals is increased by either improving the objective fitness $F_{i}$ or reducing the number of network perturbations $I_{i}$. Hence, there is a need to define which increase in $F_{i}$ justifies the experimental effort of adding another reaction or gene deletion. To control the trade-off between the reduction of simultaneous genetic interventions and the maximization of target product yield, we introduced the trade-off factor y and extended Equation (8) yielding:

(9) $${\hat{F}}_{i}=F_{i}+F_{i}\cdot y\cdot \left(I_{max}-I_{i}\right),$$

where y≥0. By increasing y, the optimization objective is shifted towards minimal perturbation sizes while the objective fitness becomes subordinated. From an economical point of view, the user can determine a minimally-necessary gain in a strain’s production fitness or capability that justifies a higher number of knockouts and, thus, an accompanied increased workload for the experimentalist. 

#### 2.3.4. Non-Native Network Edge Insertions

Inspired by the OptStrain and SimOptStrain frameworks [8,11], we expanded the basic GA to identify non-native reaction insertions while simultaneously searching for a set of reaction or gene deletions, which, in combination, maximize overproduction. We particularly focused on novel network edges and, hence, confined the set of possible insertion targets to reactions that act on metabolites inherent to the wild-type model only. Respective candidate reactions were derived and curated by consulting the MetaNetX [40], BiGG [42], eQuilibrator [43], and KEGG [44] databases to create a databank model providing a repertoire of possible novel functionalities to the GA (cf. Supplementary I.3).

### 2.4. Analysis of the Evolution of Populations

#### A Measure of Population Diversity: The Hamming Distance

The average Hamming distance between pairs of individuals can be used to quantify the diversity of a population, which aids in investigating the time convergence of GAs [36,45]. The Hamming distance counts the number of differing bits in two individuals, hence, for all possible pairs of individuals a population’s average Hamming distance is calculated by:

(10) $$HD=\frac{\sum_{i}^{N_{Pa}}\sum_{j}^{N_{B}}\left|B_{j}^{i1}-B_{j}^{i2}\right|}{N_{Pa}\cdot HD_{max}},$$

with:

(11) $$N_{Pa}=\frac{N_{P}}{2}\left(N_{P}-1\right)$$

In Equation (10), $B_{j}^{i1}$ is the *j*th bit of the first individual of the *i*th pair in the population. Additionally, HD is normalized by the maximally possible Hamming distance between two individuals. Therefore, we will generally use HD for the normalized, average Hamming distance in this work. Note that Equation (11) represents a formula to calculate the number of possible pairs from $N_{P}$ individuals of a population.

### 2.5. Metabolic Model Preprocessing

In this work, the *E. coli* K-12 MG1655 core [46], as well as the genome-scale reconstruction *i*JO1366 [47], were employed. Preceding any GA optimization, a model compression was conducted by eliminating sink and source reactions, which consume or produce unbalanced metabolites. Therefore, reactions that could not carry any flux were iteratively identified by flux variability analysis and subsequently deleted. When gene deletion targets are considered, genes being connected by an AND operator in the same GPRs were lumped. For example, genes encoding for sub-units that are found in only one particular enzyme were considered as one gene.

Additionally, the deletion target space was reduced to minimize the complexity of the optimization problem. Partly following the protocol of Feist et al. [23], reactions not associated to any genes, such as spontaneous, diffusion, and exchange reactions, were not considered as deletion targets. Furthermore, all transport reactions as well as reactions being involved in cell envelope biosynthesis, membrane lipid metabolism, murein biosynthesis, tRNA charging, and glycerophospholipid metabolism were removed from the target space.

### 2.6. General Conduct for the Application of the Genetic Algorithm

All simulations employing the *E. coli* core and the genome-scale *i*JO1366 model were replicated five and three times, respectively. All data shown is an average of the replicates and given errors denote the correspondent standard deviation. The GA was implemented in MATLAB 2016b (The Mathworks, Inc., Natick, MA, USA) and is freely available on GitHub (https://github.com/Spherotob/GAMO_public). All computations and the evaluation of the results were conducted on a Windows 7 machine with 16 GB of RAM and an AMD FX-8350 Eight-Core (@ 4.00 GHz) processor.

## 3. Results

### 3.1. GA Parameter Sensitivity Analysis

The performance of GAs on arbitrary optimization problems is strongly dependent on the GA parameters and a sound setting is generally hard to predict. It is, thus, advisable to conduct a thorough parameter sensitivity analysis for a specific problem class to derive the most advantageous parameter ranges. Therefore, we performed a parameter sensitivity analysis for a basic GA (cf. Section 2.1) on strain design problems using the *E. coli i*AF1260 core metabolic reconstruction. We consistently employed the BPCY as fitness function, which was calculated according to the descriptions given in Section 2.1.2. For an overview of the used GA parameters for each conducted simulation in this work and the obtained best intervention strategies we refer to Supplementary File 4 and 5.

#### 3.1.1. Mutation Rate

**Figure 2:**
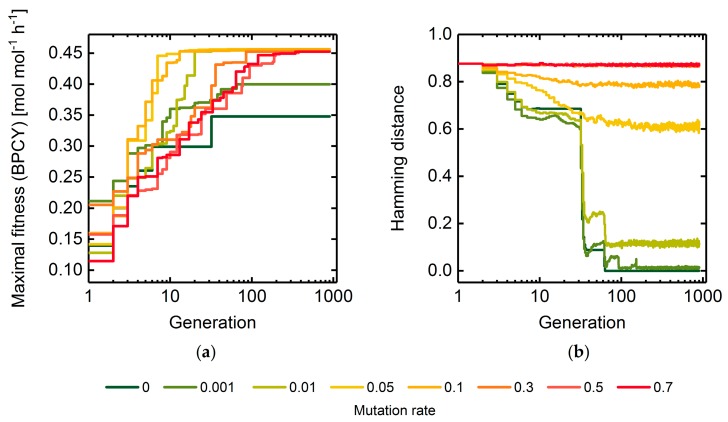
Maximal fitness (**a**) and hamming distance (**b**) across the populations of every thread in each generation using mutation rates between 0 and 0.7. Deletion of maximally five reactions were allowed while using succinate BPCY as the engineering objective. Hamming distance progressions for mutation rates 0.5 and 0.7 overlap each other.

**Figure 3:**
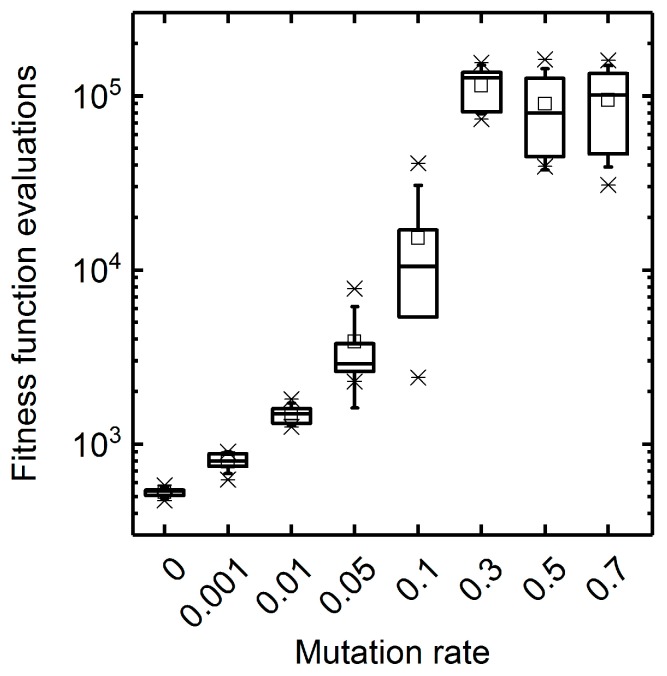
Number of fitness function evaluations until maximal final fitness was reached. Box plots represent five replicate GA runs applying the respective mutation rate. Succinate BPCY was used as the engineering objective.

The arbitrary mutation of individuals is a central operator of GAs driving the exploration of the solution space for globally optimal solutions. At low mutation rates, the search of GAs is narrowed to the local surroundings of a population’s individuals, which is likely to lead to premature convergence. On the contrary, too high mutation rates diminish the fitness intensification in the local area of a population and, thus, convert GAs into random search methods, which results in low convergence speeds. This is illustrated by the maximal fitness and the Hamming distance trends when optimizing for succinate biomass-product coupled yield (BPCY) using a basic GA at different mutation rates (Figure 2). Evolution of individuals stopped at a relatively low, suboptimal fitness value after approximately 40 generations for low mutation rates up to 0.001 due to a vanishing population diversity. Contrarily, at elevated mutation rates above 0.3, the convergence to optimal fitness values was slow and the diversity remained at high levels without exhibiting any indications of intensification. A mutation rate of 0.05 exhibited an advantageous compromise between exploration and intensification of the target space and, thus, led to the highest convergence rates. As shown in Figure 3, fast convergence correlated with low numbers of fitness function evaluations needed to reach maximal fitness and, thus, to low computational costs. Mutation rates below 0.01 exhibited the lowest computational costs but impeded finding the optimal solution with a fitness of $0.46\;{\mathrm{mol}\;\mathrm{mol}}^{-1}{\;h}^{-1}$. If not stated otherwise, we used a mutation rate of 0.05 in all further simulations to reasonably limit the number of fitness function evaluations during GA runs while avoiding a radical drop in population diversity and, thus, premature convergence. Anticipating the optimization of the selection rate (cf. Section 3.1.2), we conducted the mutation rate sensitivity analyses at selection rates 0.25 (Supplementary Figure S2) as well as 0.75 (Supplementary Figure S3) and found no qualitative differences to the presented results at a selection rate of 0.5. We conclude that an optimal choice for the mutation rate is not affected by the selection rate and can be optimized sequentially. Moreover, we arrived at the same conclusions for the population size by also conducting mutation rate sensitivity analyses at population sizes of 10 (Supplementary Figure S4) and 50 (Supplementary Figure S5).

Fixing the mutation rate during GA runs was previously shown to be superior to variable, adaptive mutation probabilities [36]. However, in contrast to the findings of Srinivas and Patnaik [36], in our simulations, adaptive probabilities generally led to a decrease in convergence speed using ethanol BPCY as engineering objective (Supplementary Figure S6). For five, seven, and ten maximal reaction deletions, we applied different ranges between the minimally- and maximally- allowable mutation rates, each centering around a mutation rate of 0.05. Intensification of solutions was more and more hampered for increasing range widths, most notable by means of static Hamming distance progressions at high levels (Supplementary Figure S6). Hence, we dismissed the promising concept of adaptive mutation probabilities for our GA formulation.

#### 3.1.2. Selection Rate and Population Size

**Figure 4:**
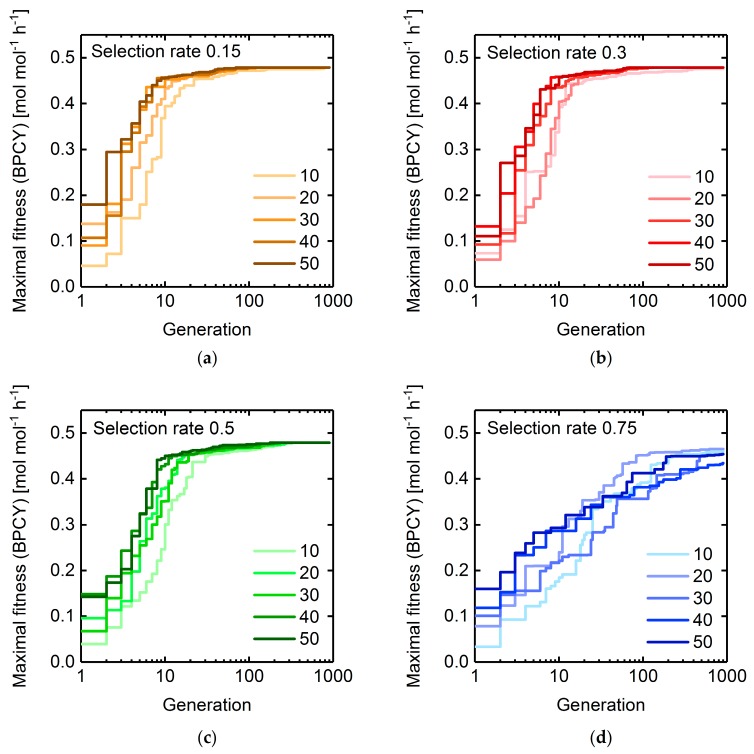
Maximal fitness progressions of GA runs using selection rates of (**a**) 0.15, (**b**) 0.3, (**c**) 0.5, and (**d**) 0.75. The color codes denote different population sizes ranging between 10 and 50. Generation numbers are plotted on a logarithmic scale. Deletion of maximally seven reactions were allowed. Succinate BPCY was used as the engineering objective.

The selection rate (specifies how many individuals or solutions of a population are selected to survive a generation) and population size (number of individuals in a population) determine how many of the fittest individuals are being selected to the mating pool for breeding new, and eventually superior, offspring individuals. Therefore, both parameters jointly influence the local search behavior of GAs in the vicinity of a population induced by the crossover operator. To assess this influence in terms of convergence characteristics and computational cost minimization, we performed GA runs with varying selection rates and population sizes using succinate BPCY as the engineering objective and limiting the intervention size to seven reaction deletions. For each tested selection rate–population size pair, the progression of the maximal fitness is shown in Figure 4.

**Figure 5:**
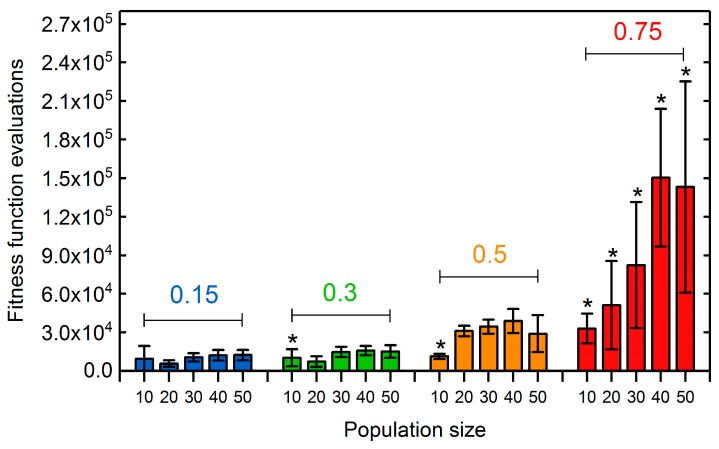
Number of fitness function evaluations until maximal final fitness was reached for GA runs applying population sizes between 10 and 50. Bars are clustered according to the employed selection rate (colored number). Error bars show the standard deviation among five replicates for each population size—selection rate pair. Asterisks denote parameter pairs with which the globally maximal fitness of $0.48\;{\mathrm{mol}\;\mathrm{mol}}^{-1}{\;h}^{-1}$ was not reached in every replicate GA run after 900 generations. Succinate BPCY was used as the engineering objective. The intervention set size was seven.

GA runs employing high selection rates of 0.75 exhibited the slowest convergence towards the maximal observed fitness of $0.48\;{\mathrm{mol}\;\mathrm{mol}}^{-1}{\;h}^{-1}$, irrespective of the chosen population size. No significant difference in the convergence behavior was observed between the lower selection rates of 0.15, 0.3, and 0.5. For specific selection rates, an increase of the population size up to 30 generally led to a faster convergence. However, significant differences became apparent in the computing time necessary to reach maximal fitness values (Figure 5). With increasing selection rates, more fitness function evaluations were required to reach the GA run specific maximal fitness. For a selection rate of 0.75, this maximal fitness did not coincide with the global maximum for any tested population size. When applying lower selection rates, non-global optima were only exhibited at a low population size of 10. Thus, a certain number of novel offspring individuals being generated at any generation had to be exceeded to provide a sufficient combinatoric for the crossover operator to effectively contribute to finding better individuals. Population sizes above 30 did not seem to significantly alter the computational cost to reach the global maximal fitness, but led to increased overall computing times and costs for a fixed number of generations (Supplementary Figure S7). Hence, for the following GA runs we chose a rather low population size of 20 to assure fast convergence characteristics while minimizing the computational burden. We decided to set the selection rate to an intermediate value of 0.25, since low selection rates may reduce computation times, but lead to a drop in the population diversity (Supplementary Figure S8), which we wanted to keep at a moderate level to maintain the exploration capability of the GA.

#### 3.1.3. Parallelization: Numbers of Generations, Gene-Flow Events, and Threads

With the parallel implementation of the GA, populations are evenly split into sub-populations, which are assigned to multiple separate processing units or workers and evolved independently from each other (Section 2.1.5). After a specified number of generations, the latest sub-populations are pooled and eventually randomly allocated again to the workers to repeat the process. Such gene-flow events (GFEs) allow for an additional mechanism to diversify populations and promote evolution towards globally optimal solutions [37].

**Figure 6:**
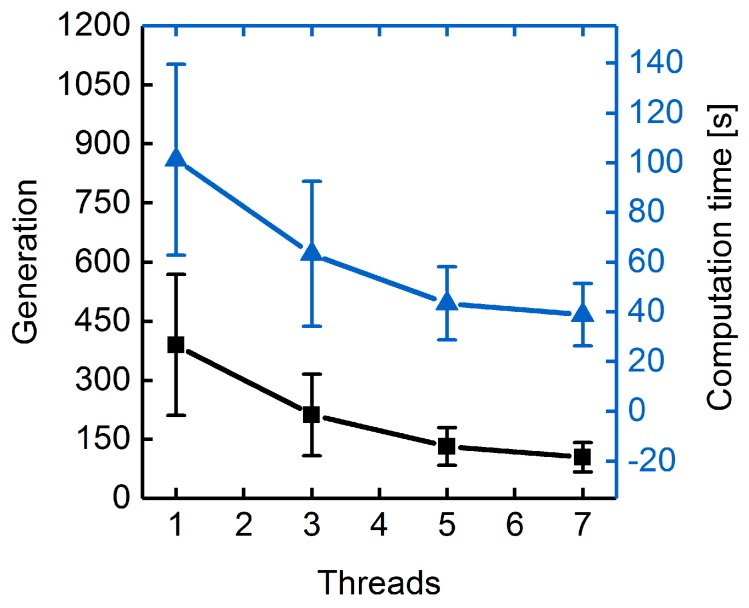
Number of generations (squares) and computation time (triangles) until maximal fitness was reached. Deletion of maximally seven reactions were allowed. Succinate BPCY was used as the engineering objective. Error bars denote the standard deviation of five replicate GA runs using one, three, five, and seven parallel threads.

**Figure 7:**
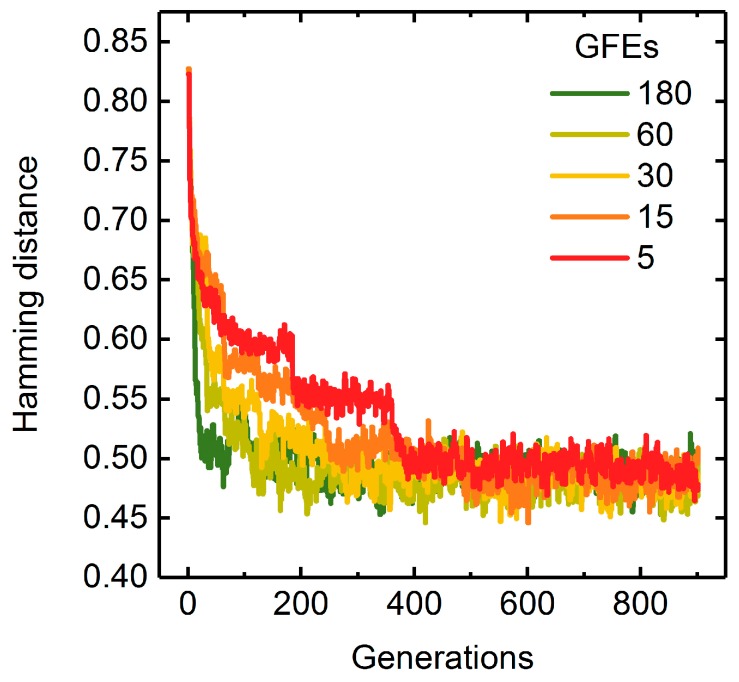
Hamming distance progressions for GA runs applying 5 to 180 GFEs while keeping the total generation number at 900. Deletion of maximally seven reactions were allowed. Succinate BPCY was used as the engineering objective. Error bars denote the standard deviation of five replicate GA runs.

Generally, parallelization of generation sequences and fitness function evaluations is applied to cut computation time, particularly when dealing with costly fitness functions [37]. GA runs using one to seven threads and searching for seven reaction deletions while applying succinate BPCY as the engineering objective showed similarly decreasing generation numbers and computation times necessary to reach the maximal fitness with increasing number of threads (Figure 6). This raises the question: How does the distribution between the number of successive generations per strand and the number of GFEs influence the GA’s performance? We tested the influence of GFEs on the performance of the GA by varying the generation size between two GFEs and the number of GFEs itself while keeping the total number of generations constant. Surprisingly, the progressions of maximal fitness at each generation suggest that changing the distribution between generation size and number of GFEs has no significant effect on the convergence behavior (Supplementary Figure S9). According to the Hamming distance, on the other hand, population diversities diminished more slowly when fewer GFEs were conducted in favor of higher generation sizes (Figure 7).

**Figure 8:**
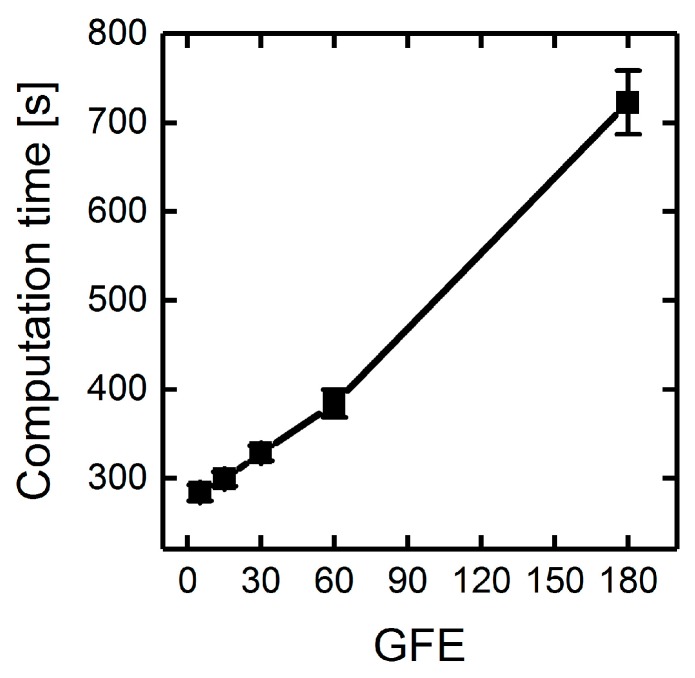
Absolute computation time of 900 generations for several pairs of GFEs and generation sizes. Deletion of maximally seven reactions were allowed. Succinate BPCY was used as the engineering objective. Error bars denote the standard deviation of five replicate GA runs.

However, the absolute computation time for the overall 900 generations is gradually reduced when decreasing the number of GFEs (Figure 8). This is mainly due to overhead computations spent on merging or splitting populations, initialization of parallel loops and distribution of data to different workers. To minimize absolute computation times and ensure appropriate population diversities throughout GA runs, we chose a generation size of 60 for the following simulations. The total number of generations was thus controlled by the number of GFEs.

### 3.2. Target Product Varieties and Minimal Intervention Set Sizes

**Figure 9:**
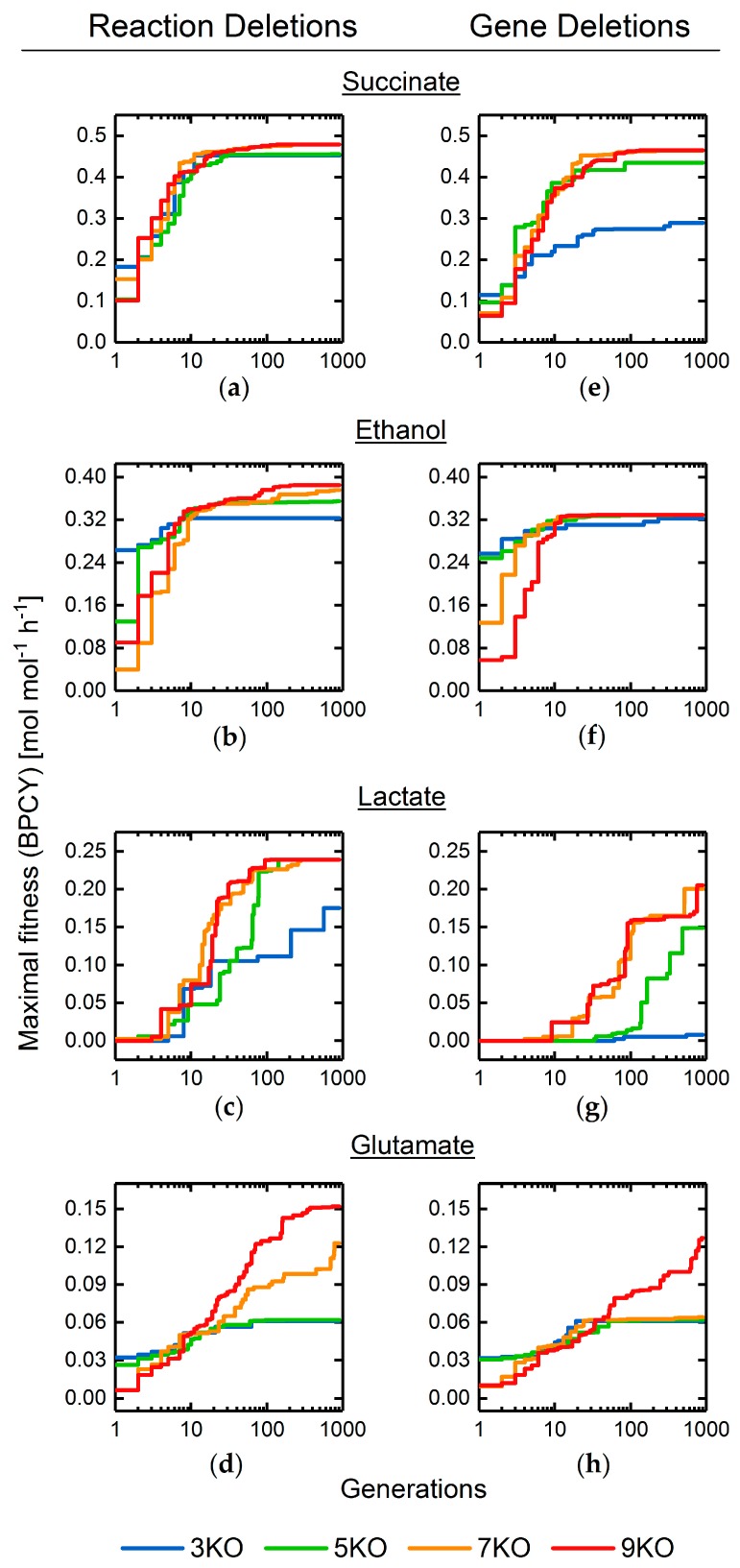
Maximal fitness progression of GA runs optimizing overproduction of succinate (**a**,**e**), ethanol (**b**,**f**), lactate (**c**,**g**), and glutamate (**d**,**h**) applying three, five, seven, and nine maximal reaction (**a**–**d**) or gene (**e**–**h**) deletions.

We used the basic GA and the optimized GA parameter set to determine strain designs for the overproduction of succinate, ethanol, lactate, and glutamate allowing maximum reaction or gene deletions between three and nine (Figure 9). Independent of the target product, the fitness for gene deletion target-sets generally converged to lower values compared with reaction target-set solutions of the same size. In all cases, the approach of the convergence region for the maximal fitness coincided with the convergence of the Hamming distance, hence, the population diversity (cf. Supplementary Figures S10 and S11).

Interestingly, the final fitness for five, seven, and nine reaction or gene deletions was the same or in the near range for all products. Hence, individuals representing a high, fixed intervention set size likely included one or more deletions, which did not contribute to the engineering objective. This is explained by our formulation of an individual (cf. Section 2.1.1), which allowed for multiple occurrences of the same target, further enforced by scaling the fitness with the number of unique targets within an individual (cf. Section 2.3.3). Accordingly, computed solutions needed to be postprocessed to extract the unique targets and actual number of deletions.

**Figure 10:**
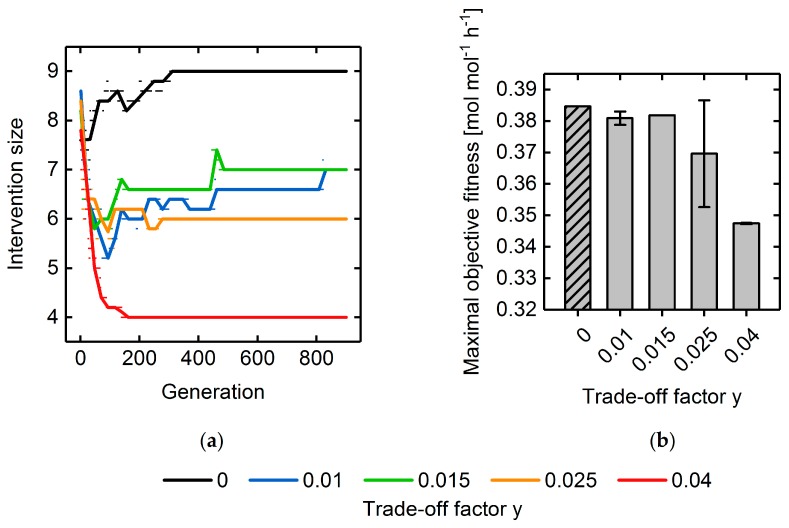
(**a**) Progressions of the intervention size of the fittest individual throughout GA runs, in which the transformed fitness function was used (cf. Equation (9)) to consider the minimization of the number of deletion targets (cf. Section 2.3.3). Values between 0 and 0.04 were employed for the trade-off factor *y* to investigate its influence on trade-off between the objective fitness and the number of simultaneous perturbations. Dots illustrate the mean intervention size over a population at a specific generation. The lines represent the corresponding linear interpolations. For the same simulations, the final objective fitness values are shown in Subfigure (**b**).

We exemplarily applied the intervention set minimization approach to ethanol overproduction using different instances of the trade-off factor y. By increasing y we were able to gradually concentrate on solutions with lower numbers of unique reaction deletions (Figure 10a). However, minimization of intervention sizes came at the expense of lowered objective fitness values and, thus, of lower ethanol overproduction capabilities (Figure 10b). For example, applying a y of 0.04 promoted quadruple deletion individuals as optimal solutions, whereas a lower y of 0.025 favored individuals with six unique reaction deletions. According to the Hamming distance and maximal fitness progressions (Supplementary Figure S12), convergence speed decreased with the increasing trade-off factor indicating that enforcement of the elimination of non-contributing deletion targets elevated the problem complexity.

### 3.3. Multi-Objective Fitness Function Optimization

To focus on the robustness of strain designs, we combined laboratory evolution-based objectives, namely gcOpt and OptKnock, as well as BPCY as a non-laboratory evolution objective, in one fitness function and identified reaction deletion strategies for the maximization of succinate, ethanol, lactate and glutamate production. A “perfect” solution would therefore guarantee a high minimal yield at any growth state while predicting an optimal compromise between growth and target synthesis rates for the deletion mutant.

**Figure 11:**
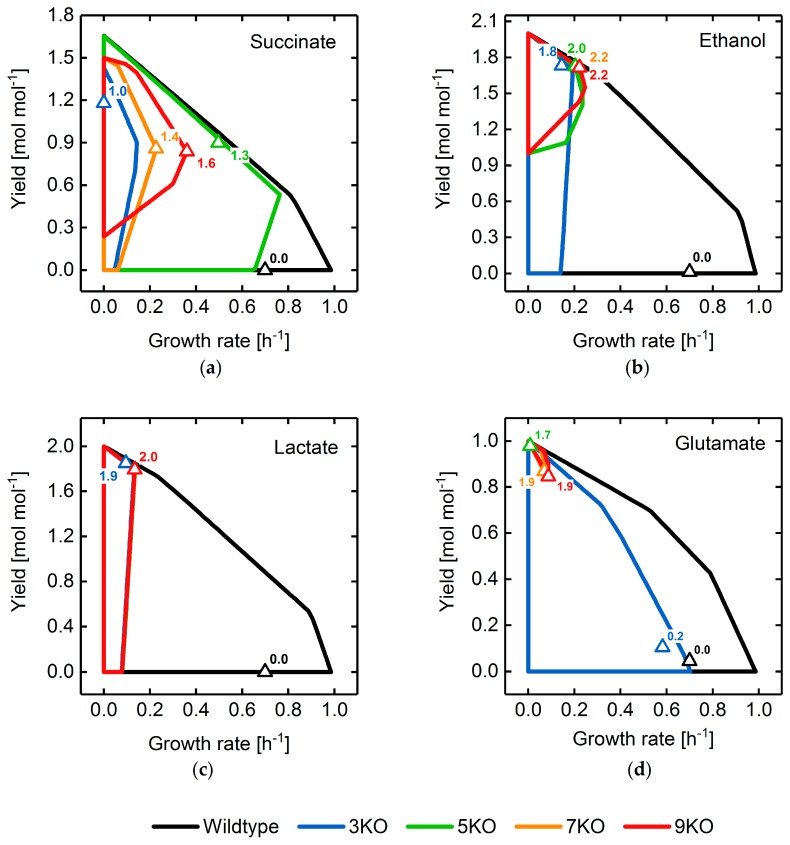
Yield spaces of wild-type, as well as mutant, *E. coli* strains optimized for the overproduction of succinate (**a**), ethanol (**b**), lactate (**c**), and glutamate (**d**) using a combination of BPCY, growth-coupling, and production rate at maximal growth rate as the engineering objective. All mutant yield spaces are based on the substrate uptake rate predicted by MiMBl. Triangles and attached numbers illustrate the phenotype prediction calculated by MiMBl and the fitness value for a strain design with a given number of reaction deletions, respectively. Refer to the Supplementary Section I.2 for a brief description of yield space calculations.

Figure 11 shows the yield spaces of GA-optimized reaction deletion mutants with succinate, ethanol, lactate, and glutamate as target products, while employing the multi-objective fitness function approach and maximal intervention set sizes between three and nine. Yields and growth rates for each mutant predicted by MiMBl are additionally illustrated. Particularly for succinate, ethanol, and glutamate, solutions were identified for which both a strong product-growth coupling and a favorable compromise between yield and growth were predicted. The latter also holds for lactate as a target, but production robustness, in terms of a guaranteed yield at any growth state, was comparably low.

Among the investigated target products, convergence characteristics of the population diversity were comparable for the same maximal allowable intervention set size (cf. Supplementary Figure S13). Moreover, they also matched the characteristics of simulations for which only the target product BPCY was used as the engineering objective.

### 3.4. Heterologous Reaction Insertion

In addition to the mere intersection of metabolic networks, simultaneous addition of non-native functionalities has been shown to further improve overproduction capabilities [11]. Using a curated databank model for the *E. coli* core model, including novel heterologous reactions (cf. Supplementary File 2), we tested the GA’s capability to identify advantageous combinations of reaction deletions and additions for the overproduction of succinate, glutamate, lactate, and ethanol. However, we refrained from introducing whole new pathways and metabolites to the wild-type organism. Rather, we limited network extensions to insertions of novel network edges to, in the context of this work, focus on the mere feasibility of integrating heterologous reaction insertions into a genetic algorithm.

**Figure 12:**
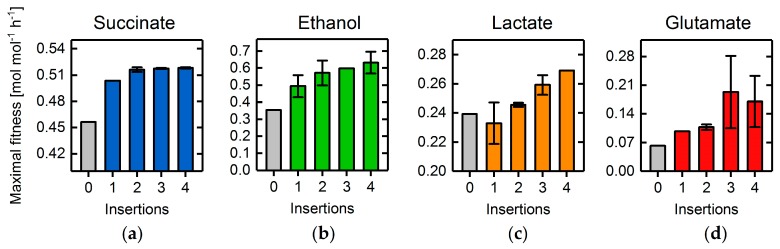
Fitness of the best individual after 1800 generations. BPCY of succinate (**a**), ethanol (**b**), lactate (**c**), and glutamate (**d**) was used as the engineering objective while applying five reaction deletions, as well as one to four novel reaction insertions. The grey bars illustrate the fitness of the best individual after 900 generations without considering any reaction additions (cf. Figure 8).

For all four target products, the GA was able to further improve the BPCY by adding between one and four novel reactions compared to corresponding quintuple deletion mutants (Figure 12). For lactate (Figure 12c) the production appears to decrease upon a single insertion. However, the relatively large standard deviation in the final fitness between the five replicate simulations indicate that higher fitness values, compared to the quintuple deletion mutant, were indeed obtained. In fact, in only one out of five simulations was the fitness significantly lower (0.21 ${\mathrm{mol}\;\mathrm{mol}}^{-1}{\;h}^{-1}$). The encountered minor difficulties in finding the global optimal solution is due to the probabilistic nature of the GA and can be avoided by increasing the number of generations. The same considerations also apply for the four and five insertions in the case of glutamate (Figure 12d), where the increased problem complexity, resulting from the higher number of insertion targets, further impeded the determination of the global optimum. In the case of succinate, replacement of the NAD^+^-dependent glyceraldehyde-3-phosphate dehydrogenase with its NADP^+^-dependent, phosphorylating counterpart (EC 1.2.1.13) and addition of an ATP-dependent citrate lyase (EC 2.3.3.8) frequently occurred in the best individuals. Simultaneously, formation of acetate and ethanol were inhibited, as well as the malic enzyme knocked out, altogether enforcing metabolic flux through the glyoxylate shunt and the reductive branch of the TCA cycle towards succinate. For the glycolytic product ethanol, switching from the NAD^+^-dependent to the NADP^+^-dependent alcohol dehydrogenase (EC 1.1.1.2) and glyceraldehyde-3-phosphate dehydrogenase (phosphorylating), as well as simultaneously deleting the NAD^+^ transhydrogenase, led to the most promising strategies. Congruently, NADH/NADPH metabolism was the preferred target for glutamate overproduction, which was spurred by the addition of the NADP^+^-dependent glyceraldehyde-3-phosphate dehydrogenase (EC 1.2.1.9) as well as the knockout of NAD^+^ transhydrogenase. The identified strain designs also suggested to increase flux through the TCA cycle by heterologous expression of the citrate oxaloacetate-lyase (EC 4.1.3.6) to recycle acetate. Interestingly, insertion of the latter in combination with the expression of the non-native NADP^+^-dependent glyceraldehyde-3-phosphate dehydrogenase and the deletion of various NADH/NAD^+^-dependent reactions also improved lactate overproduction.

### 3.5. Increasing the Complexity and Predictive Power of Employing Genome-Scale Models

Genome-scale network reconstructions represent dense information sources of the current knowledge about microbial metabolic functionalities. In combination with constraint-based modeling approaches they can aid in thoroughly predicting the behavior of microbes and their response to genetic perturbations [6]. The sheer size and complexity of genome-scale models (GEM), however, drastically increase the computational burden for in silico strain design methods and eventually render their application infeasible. GAs on the other hand are particularly suited for handling costly fitness functions and are able to provide at least near-optimal solutions for large-scale optimization problems [36,37].

**Figure 13:**
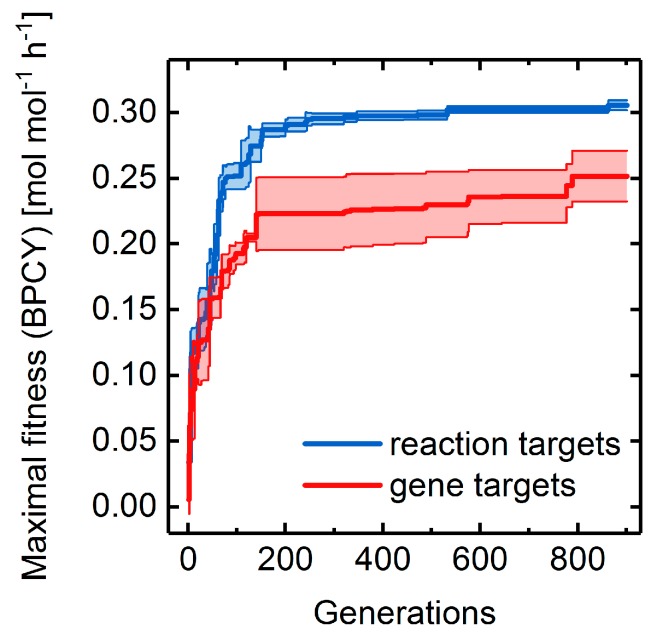
Fitness progressions of GA runs optimizing succinate overproduction in the *E. coli i*JO1366 model applying gene (red line) and reaction (blue line) deletions. Standard deviation among three replicate GA runs are illustrated as error bands.

We applied the basic GA framework to identify quintuple gene and reaction deletion strategies that maximize succinate production using the *E. coli* GEM *i*JO1366. Similar to the *E. coli* core model (cf. Section 3.2), fitness converged slower and the final maximal fitness was lower when searching for gene, rather than reaction, targets (Figure 13). In contrast, the maximally observed succinate BPCYs decreased by 33% for reaction targets and 42% for gene targets, pointing to a potentially misleading oversimplification of the metabolic repertoire in the *E. coli* core model for predicting exact mutant phenotypes. However, fitness convergence was unexpectedly fast for the genome-scale model, despite its large target space. Therefore, we are confident that the optimized GA parameter set resulting from the undertaken parameter analyses are optimal for any stoichiometric metabolic model.

**Figure 14:**
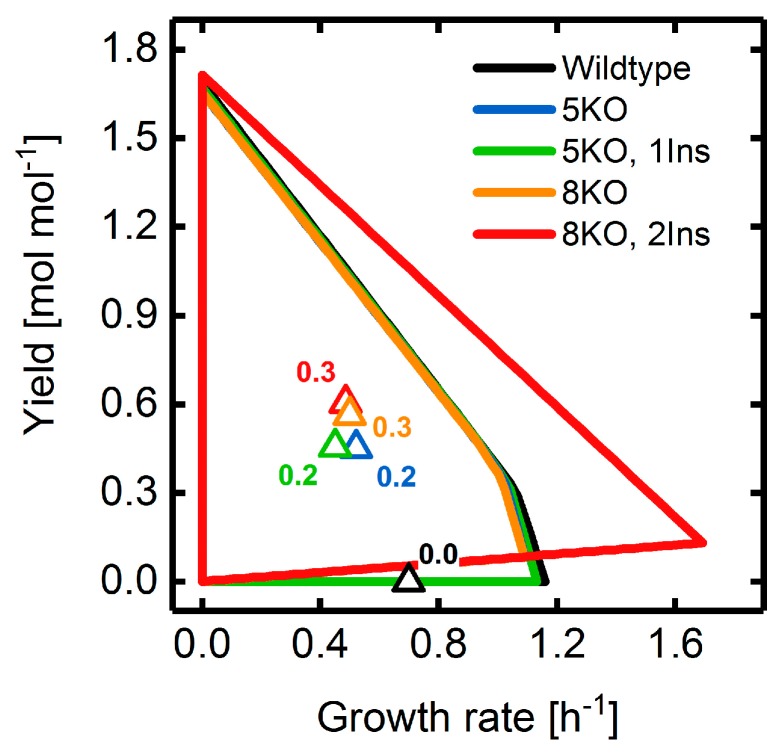
Yield spaces of wild-type, as well as mutant, *E. coli* strains optimized for the overproduction of succinate using a combination of BPCY, GCS, and production rate at a maximal growth rate as the engineering objective. The legend shows the maximal allowable numbers of gene deletions (KO) and reaction insertions (Ins). All mutant yield spaces are based on the substrate uptake rate predicted by MiMBl. Triangles and attached numbers illustrate the phenotype prediction calculated by MiMBl and the corresponding BPCY, respectively. Note that yield spaces of the 5KO and 8KO mutant overlap with each other. Refer to the Supplementary Section I.2 for a brief description of yield space calculations.

To exploit the full potential of the advanced GA, succinate overproducing strain designs were identified applying a multiple objective fitness function while minimizing the intervention set size using a *y* of 0.1. Figure 14 shows the yield spaces and predicted yields for four strain designs comprising different numbers of gene deletions and reaction insertions (cf. Supplementary File 3 for the non-native network edges of *i*JO1366). All strategies with five gene deletions shared a predicted BPCY of $0.2\;{\mathrm{mol}\;\mathrm{mol}}^{-1}{\;h}^{-1}$ compared to $0.3\;{\mathrm{mol}\;\mathrm{mol}}^{-1}{\;h}^{-1}$ of the octuple deletion and double insertion mutant. Both the octuple deletion and the quintuple deletion-only strain designs exhibited a slightly holistic growth-coupling, i.e., biomass yields above zero for all accessible growth states [41]. In contrast, the first showed a significant extension of the yield space up to a maximal growth rate of $1.7\;h^{-1}$, due to the additional insertion of the quinate dehydrogenase (EC 1.1.1.282) to the shikimate pathway. This is the result of an elevated NADPH synthesis rate, which was similarly observed in other, sub-optimal strain designs where, e.g., the NADP^+^-dependent glyceraldehyde-3-phosphate dehydrogenase (EC 1.2.1.13) was inserted. However, insertion of novel functionalities did not significantly improve succinate overproduction as compared to the deletion-only strain designs and in case of the quintuple deletion mutants even showed lowered fitness values (Supplementary Figure S14a). Presumably, novel network edges are not of critical concern for optimizing succinate production in *E. coli*. Identification of significantly better strain design solutions at elevated generation numbers is also unlikely, since the population diversities reached plateau regions indicating approaching fitness convergence (Supplementary Figure S14b). Only for the octuple deletion and double insertion cases did a drop in the Hamming distance approximately from generation 1600 onward suggest incomplete convergence.

Interestingly, the final best strain designs always contained fewer perturbations as was maximally possible, which is illustrated by the higher final fitness values compared to the objective fitness values (Supplementary Figure S14a). For example, the search for overproduction mutants with five gene deletions and one reaction insertion led to a triple, instead of a quintuple, deletion strain design being a good compromise between the number of perturbations and objective fitness. Due to the minimization of intervention size approach, the reduced intervention set size was favored at the expense of a narrowed yield space in contrast to the full intervention potential.

One main overproduction principle, however, was the enforcement of flux through the glyoxylate shunt by deleting the fumarase genes Δ*fumA*, Δ*fumB*, and Δ*fumC*. Moreover, an increase of the anaplerotic phosphoenolpyruvate carboxylase reaction by a knockout of the NAD^+^-dependent malate dehydrogenase (Δ*mdh*) or pyruvate kinase (Δ*pykA*, Δ*pykF*) occurred frequently, presumably due to the elevated recapture of carbon dioxide. For the complete strategies we refer to the Supplementary Information.

## 4. Discussion

By simultaneously incorporating previously published, as well as novel, engineering approaches into a basic GA framework, we could demonstrate the versatility and broad applicability of GAs for solving strain design problems. The addition of novel reactions and functionalities, in consideration of gene, as well as reaction, deletions, application of multiple optimization objectives, and minimization of necessary network perturbations proved to be simultaneously manageable by the GA. Such an integrative approach allows for an increased level of robustness in terms of overproduction stability and efficiency of mutant strain designs, as well as the consideration of practicability of necessary genetic interventions.

In light of ongoing refinements of purely stoichiometric models to enhance their predictive power by introducing novel, kinetics-related protein or enzyme expression constraints [48,49], or whole gene expression systems [35], GAs were shown to be able to handle the accompanied increase in model and prediction method complexity in this work. Due to the relatively straightforward implementation of GAs, biological and engineering objectives can be readily adapted to specific questions, applications, and requirements.

The performance of the GA is, in any case, strongly dependent on the balance between a broad diversity in the genetic pool of consecutive populations and the focusing to the most fit solutions or individuals. An exaggerated concentration on the local search characteristic of the GA, e.g., by applying small mutation rates, led to premature convergence to non-optimal solutions, which became apparent by a drastic drop in the population diversity. On the other hand, constantly high diversities indicated a strong exploration of the solution space, but were accompanied by slow convergence rates and a high computational effort necessary to identify optimal solutions. GA parameters were thus identified by a parameter analysis to blend both exploration, as well as intensification characteristics, and guarantee good optimization performances for any strain design problem. Thus, we reduced the chance for premature convergence and simultaneously minimized the computing time. Above all, the favorable convergence characteristics were not affected when we applied more complex engineering objectives, such as a multi-objective fitness function.

Avoiding premature convergence is particularly important if, in future developments, convergence may be detected at runtime to terminate the GA and output the optimal solution instantaneously. A stagnation of the population diversity, which is quantified by the Hamming distance of a population, can potentially serve as a convergence criterion. However, if a population converged to a global or a local optimum can hardly be decided and, thus, premature convergence needs to be circumvented in the first place. Therefore, slight promotion of the GA’s exploration characteristics by careful parameter adaptions can be advantageous. 

In a previous study we claimed that strong growth-coupling is effectively generated by the perturbation of cofactor balancing and ATP, in particular [41], which, however, may be too metabolically destructive. Congruent to those findings, strain designs identified in this work, which strongly coupled succinate, ethanol, or glutamate production to growth, incorporated, among others, the knockout of the ATP synthase, thus shifting the ATP supply to the substrate phosphorylation level. Simultaneously, high BPCYs were predicted for these strain designs strengthening the applicability of such ATP-restricting engineering approaches to gain robust overproduction strains.

The addition of novel metabolic reactions taken from a model databank generally targeted the cofactor and, particularly, the NADH/NADPH metabolism, besides the inhibition of byproduct formation. A common strategy was to replace NAD^+^-dependent reactions with their NADP^+^-dependent counterparts, while simultaneously deleting the NADH dehydrogenase, NAD^+^ transhydrogenase or other NADH-dependent reactions, as was also previously suggested for succinate overproduction by Kim et al. [11] based on their findings employing SimOptStrain. However, by exploiting the *E. coli i*JO1366 GEM and the full capacity of the GA’s features, we identified a rather different strain design compared to other theoretical or experimental studies. Whereas it has been frequently suggested to directly suppress byproduct formation, e.g., by knocking out *ackA*, *ldhA*, or *pfl* [50], the GA framework applying an *E. coli* GEM predicted a redirection of the TCA cycle flux towards the glyoxylate shunt to be most beneficial for succinate production. Moreover, and in line with results from the core metabolic model, reduction of NADH generation in favor of NADPH appeared to be a key design principle. This was pronounced by the suggestion to include the non-native NADP^+^-dependent glyceraldehyde-3-phosphate dehydrogenase or quinate dehydrogenase, which significantly improved theoretical maximal growth. Since our design suggestions resulted from the simultaneous application of various engineering objectives, a comprehensive metabolic model, and the consideration of actual gene-protein dependencies, as well as detailed wild-type metabolic flux data, it offers the most reliable basis for experimental transfer. Thus, we are looking forward to further investigating or even practically applying the presented designs, which, however, was out of the scope of this work.

In summary, we could demonstrate that simultaneous application of multiple, complex engineering objectives to genome-scale metabolic models for strain design purposes is indeed feasible using GAs. Moreover, GAs offer the potential to integrate even more complex objectives and methods, and their performance may be tuned according to highlighted characteristics and parameter sensitivities.

## Supplementary Materials

The following are available online at http://www.mdpi.com/2218-1989/8/2/33/s1. The Genetic Algorithm for Metabolic Engineering (GAMO) framework developed and used in this work is freely available on GitHub (https://github.com/Spherotob/GAMO_public, doi:10.5281/zenodo.1208048). Supplementary File 1: Section I.1.Determination of reference flux distributions. Section I.2. A simplified calculation of the growth-coupling strength. Section I.3. A databank model including novel network edges. Figure S1. Scheme of a wild-type yield space showing no growth-coupling and a growth-coupled strain design. Figure S2. Maximal fitness and Hamming distance progressions of GA runs for the mutation rate sensitivity analysis using a selection rate of 0.25. Figure S3. Maximal fitness and Hamming distance progressions of GA runs for the mutation rate sensitivity analysis using a selection rate of 0.75. Figure S4. Maximal fitness and Hamming distance progressions of GA runs for the mutation rate sensitivity analysis using a population size of 10. Figure S5. Maximal fitness and Hamming distance progressions of GA runs for the mutation rate sensitivity analysis using a population size of 50. Figure S6. Maximal fitness and Hamming distance progressions applying an adaptive mutation probability approach for a basic genetic algorithm. Figure S7. Total number of fitness function evaluations after 900 generations using a basic genetic algorithm and applying different selection rates and population sizes. Figure S8. Hamming distance progressions for GA runs using selection rates between 0.15 and 0.75. Figure S9. Maximal fitness progressions applying different numbers of gene flow events at constant numbers of total generations. Figure S10. Maximal fitness and Hamming distance progressions using a basic genetic algorithm and the *E. coli* core model to identify reaction deletions for succinate, ethanol, lactate, and glutamate overproduction. Figure S11. Maximal fitness and Hamming distance progressions using a basic genetic algorithm and the *E. coli* core model to identify gene deletions for succinate, ethanol, lactate, and glutamate overproduction. Figure S12. Maximal fitness and Hamming distance progressions of genetic algorithm runs using a minimization of intervention set size approach. Figure S13. Hamming distance progressions for genetic algorithm runs using multiple objective functions simultaneously. Figure S14. Maximal fitness and Hamming distance progressions for genetic algorithm runs using the *E. coli* genome-scale model *i*JO1366. Table S1. Minimally- and maximally-expected, as well as standard intracellular concentrations, of gaseous metabolites. Supplementary File 2. Non-native network edges for the *E. coli i*AF1260 core model identified following the descriptions in the Supplementary text. Supplementary File 3. Non-native network edges for the *E. coli i*JO1366 genome-scale model identified following the descriptions in the Supplementary text. Supplementary File 4. Genetic algorithm parameter sets used in each conducted simulation in this work. Supplementary File 5. Collection of all relevant, identified strain designs.

## Nomenclature

**Symbol**	**Explanation**
B	A bit in the binary representation of an individual
$\hat{F}$	Intervention size-scaled fitness
F	Objective fitness
$F_{R}$	Fitness of the best discarded individual
μ	Specific growth rate
$N_{B}$	Number of bits per individual
$N_{D}$	Number of interventions per individual
$N_{G}$	Number of subsequent generations
$N_{GFE}$	Number of subsequent gene flow events
$N_{P}$	Population size
$N_{Pa}$	Number of possible pairs of individuals
$N_{S}$	Number of selected individuals
$N_{T}$	Number of target reactions
R	Mutation rate
$v_{P}$	Production rate
$v_{S}$	Substrate uptake rate
X	Selection rate
y	Trade-off factor
