# Supplementary material for: Genetic Optimization Algorithm for Metabolic Engineering Revisited
Source: Metabolites. 2018 May 16;8(2):33. doi: 10.3390/metabo8020033 (PMC6027426; doi:10.3390/metabo8020033)
Supplement: Supplementary file 1 [file metabolites-08-00033-s001.zip › Supplementary Files/Supplementary File 1_GAMO.pdf]

metabolites

Supplementary Materials: Genetic Optimization Algorithm for Metabolic Engineering Revisited

Tobias B. Alter [1], Lars M. Blank [2] and Birgitta E. Ebert *

[1] tobias.alter@rwth-aachen.de
[2] lars.blank@rwth-aachen.de
* Correspondence: birgitta.ebert@rwth-aachen.de; Tel.: +49 241 80 266 48

I. Supplementary Text

I.1 Determination of Reference Flux Distributions

To apply MiMBl for determining mutant strain phenotypes, a reference or wildtype flux distribution is crucial. We derived individual reference flux distributions for both the *E. coli* core and genome-scale model from appropriate intra- and extracellular metabolic flux data by subsequently solving two linear optimization programs. Firstly, we minimized the sum of the absolute difference between simulated and experimentally determined fluxes. Secondly, while fixing the objective function of the former linear program to its optimal value, the sum of all other absolute flux values in the model, excluding those of exchange reactions, was minimized. This method follows the descriptions of Long et al. [1]. We used central carbon metabolism flux data of an aerobic chemostat cultivation at a dilution rate of $0.7\ h^{-1}$ taken from Ishii et al. [2].

I.2 A Simplified Calculation of the Growth-Couling Strength

The original representation of the growth-coupling strength (GCS) is based on a quantitative comparison of the yield space areas between the mutant and wildtype strain [3]. Yield spaces are generated by consecutively solving growth rate minimization as well as maximization problems for the whole range of accessible product yield states. As illustrated in Figure S1, the area below the lower yield bound (IA) and the total area under the upper yield hull curve of a strain design are used

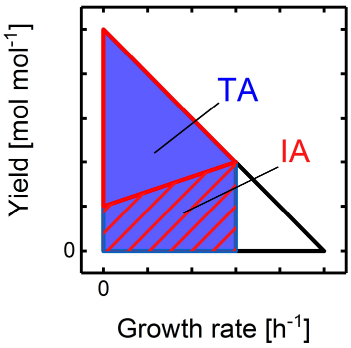

Figure S1: Scheme of a wild-type yield space showing no growth-coupling (black hull curve) and a growth-coupled strain design (red hull curve). The blue area TA illustrates the yield space of the wild-type up to the maximal growth rate of the mutant strain. The inaccessible yield space IA below the lower yield bound of the mutant is marked by the red hatched area. Figure adapted from Alter et al. 2018 [3].

to deduce the GCS. The ratio between the minimally guaranteed product yield $Y_{min}^{\mu_{max}}$ at maximal growth and the theoretical maximal yield Y_{max} are additionally incorporated in the calculation of the GCS to also consider the maximal production capabilities of mutant strains. Hence, GCS is defined by Equation 1:

$$GCS = \frac{IA}{TA} \cdot \frac{Y_{min}^{\mu_{max}}}{Y_{max}} \quad (1)$$

Since the exact determination of the yield space areas is computationally expensive, calculation of the GCS had to be simplified for a utilization as an engineering objective for the GA. Therefore, we derived an approximation using only two distinct points on the envelope edge, which are computed by two separate linear programs. These yield the target production rate $v_t^{\mu_{max}}$ at maximal growth rate μ_{max} and the minimally guaranteed production rate $v_t^{\mu_{red}}$ at $\mu_{red} = 0.9 \cdot \mu_{max}$. We used the linear extrapolation of both points as an indicator for the growth-coupling characteristics. Therefore, the production rate axis intercept of the extrapolation was determined by Equation 2:

$$v_{t,i} = \frac{\frac{v_t^{\mu_{max}}}{\mu_{max}} - \frac{v_t^{\mu_{red}}}{\mu_{red}}}{\frac{1}{\mu_{max}} - \frac{1}{\mu_{red}}}, \quad (2)$$

A $v_{t,i} \leq 0$ indicates weak growth-coupling and the area below the approximated lower production rate bound A_{weak} was calculated by

$$A_{weak} = 0.5 \cdot v_t^{\mu_{max}} \cdot \left(\mu_{max} + \frac{\mu_{max} \cdot v_{t,i}}{v_t^{\mu_{max}} - v_{t,i}}\right). \quad (3)$$

If $v_{t,i}$ is positive, the strain design exhibits strong growth-coupling and A_{strong} is

$$A_{strong} = \left(\mu_{max} \cdot v_t^{\mu_{max}}\right) - \left(0.5 \cdot \mu_{max} \cdot \left(v_t^{\mu_{max}} - v_{t,i}\right)\right). \quad (4)$$

To derive the GCS approximation, the area of the wildtype production rate beyond μ_{max} is added to A_{weak} or A_{strong} and the sum is normalized by the total area of the wildtype production envelope A_{wt}, hence

$$GCS = \frac{A_{weak/strong} + \left(0.5 \cdot v_t^{\mu_{max}} \cdot (\mu_{max}^{wt} - \mu_{max})\right)}{A_{wt}}, \quad (5)$$

where μ_{max}^{wt} is the theoretical maximal growth rate of the wildtype strain.

I.3 A Databank Model Including Novel Network Edges

The establishment of a databank model comprising novel functionalities or reactions was inspired by the OptStrain [4] and SimOptStrain [5] framework. In this work, however, we focused on novel network edges only, meaning that all metabolites of non-native reactions in the database model had to be present in the wildtype metabolic model. Using the following protocol, we searched or used the MetaNetX [6], BiGG [7] and KEGG [8] databases for reactions that fulfill these constraints. The protocol also includes curation steps to determine reaction directionalities based on thermodynamic data taken from the eQuilibrator database [9], thereby guaranteeing correct reaction mass and charge balances.

1. Load the complete MetaNetX database for biochemical reactions and metabolites and match their identifiers with the BiGG database namespace.
2. Discard reactions with dubious mass balances according to MetaNetX and those already present in the wildtype metabolic model.
3. Discard reactions that act on metabolites not present in the wildtype metabolic model.

4. Determine directionality of reactions according to the standard Gibbs Free Energy of Reaction $\Delta_r G'^{\circ}$ at pH 7 and an ionic strength of $0.1\,M$. If $\Delta_r G'^{\circ}$ cannot be provided for a reaction, it is assumed to be reversible and for all other reactions:
   a. Extract $\Delta_r G'^{\circ}$ and its standard deviation from the eQuilibrator database.
   b. Calculate a physiologically relevant Gibbs Free Energy of Reaction $\Delta_r G'^{m}$ using standard concentrations of $1\,mM$ for each participating metabolite. Water and protons do not contribute to $\Delta_r G'^{m}$. Standard concentrations for oxygen, carbon dioxide and hydrogen are listed in Table S1. For a general reaction of the form $\nu_i s_i + \nu_j s_j \leftrightarrow \nu_k p_k + \nu_l p_l$ with educts s_i and s_j, the products p_k and p_l as well their respective stoichiometric coefficients ν, $\Delta_r G'^{m}$ is calculated to:

$$\Delta_r G'^{m} = \Delta_r G'^{\circ} + RT \ln\left(\frac{c_{p_k}^{\nu_k} \cdot c_{p_l}^{\nu_l}}{c_{s_i}^{\nu_i} \cdot c_{s_j}^{\nu_j}}\right), \tag{6}$$

   where c is the molar concentration of a metabolite specified by the indices and $R = 8.314\,J\,K^{-1}\,mol^{-1}$ the universal gas constant. Furthermore, we assume a standard temperature $T = 298.15\,K$. For $\Delta_r G'^{m}_{max}$, minimal and maximal expected standard concentrations are used for the educt and product metabolites, respectively. This is reversed for the calculation of $\Delta_r G'^{m}_{min}$.
   c. Determine the maximal deviation from $\Delta_r G'^{m}$. Therefore, if $\Delta_r G'^{m} < 0$, calculate a maximally expected Gibbs Free Energy of Reaction $\Delta_r G'^{m}_{max}$ following the descriptions in b) and Equation 1. In this case, use a maximally expected concentration of $20\,mM$ for all products and a minimally expected concentration of $10^{-9}\,mM$ for all educts. Maximal and minimal concentrations of oxygen, carbon dioxide and hydrogen are listed in Table S1. Likewise, if $\Delta_r G'^{m} > 0$, $\Delta_r G'^{m}_{min}$ is instead calculated using maximally and minimally expected concentrations for the educts and products.
   d. Compare $\Delta_r G'^{m}_{max/min}$ and $\Delta_r G'^{m}$. If both $\Delta_r G'^{m}_{max/min}$ and $\Delta_r G'^{m}$ have the same sign, the reaction is labelled irreversible in the direction according to the sign. A reaction is also treated as irreversible if $\Delta_r G'^{m}_{max/min}$ and $\Delta_r G'^{m}$ exhibit differing signs and the relation $\left|\Delta_r G'^{m}_{max/min}\right| / \left|\Delta_r G'^{m}\right|$ does not exceed a value of 0.4. In all other cases, particularly if $\left|\Delta_r G'^{m}_{max/min}\right| > \left|\Delta_r G'^{m}\right|$, a reaction is reversible.
5. Check mass and charge balances of reactions according to the atomic compositions of educts as well as products and correct for missing protons or water molecules.

The selected and curated reactions are integrated in a database model and flux bounds are chosen according to the identified reaction directionalities.

II. Supplementary Figures and Tables

Table S1: Minimally and maximally expected as well as standard intracellular concentrations of gaseous metabolites.

Metabolite	Minimally expected concentration [mM]	Maximally expected concentration [mM]	Standard concentration [mM]
Oxygen	10^{-11}	0.055	0.0275
Carbon dioxide	10^{-11}	1.4	0.7
Hydrogen	10^{-11}	0.034	0.017

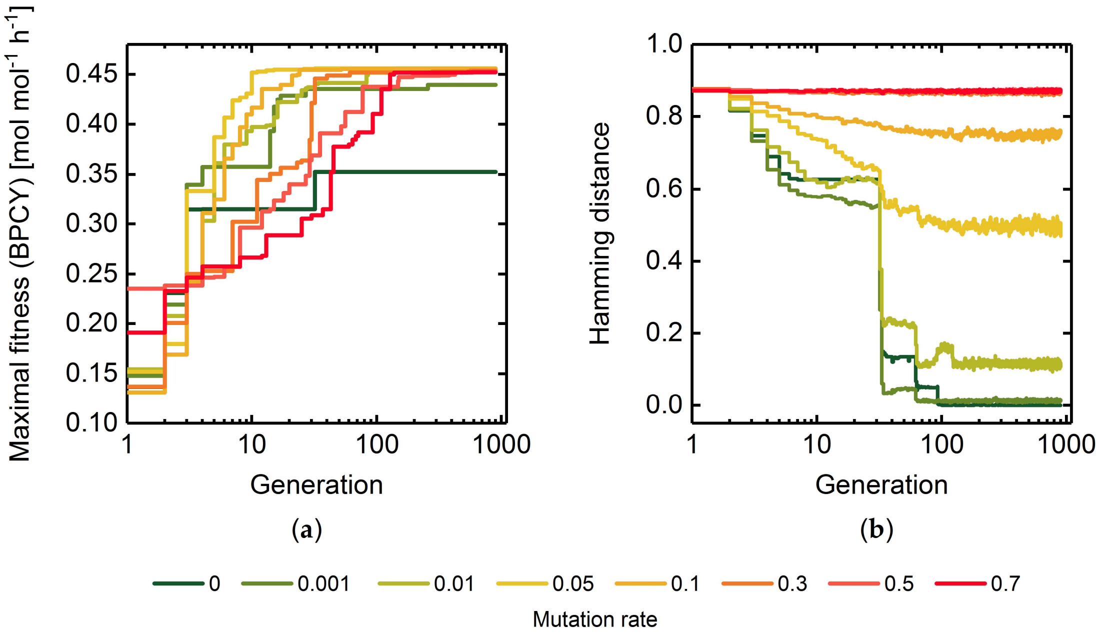

Figure S2: Maximal fitness (**a**) and hamming distance (**b**) across the populations of every thread in each generation using mutation rates between 0 and 0.7. Deletion of maximally five reactions were allowed while using succinate BPCY as the engineering objective. Hamming distance progressions for mutation rates 0.5 and 0.7 overlap each other. Here, a selection rate of 0.25 was employed.

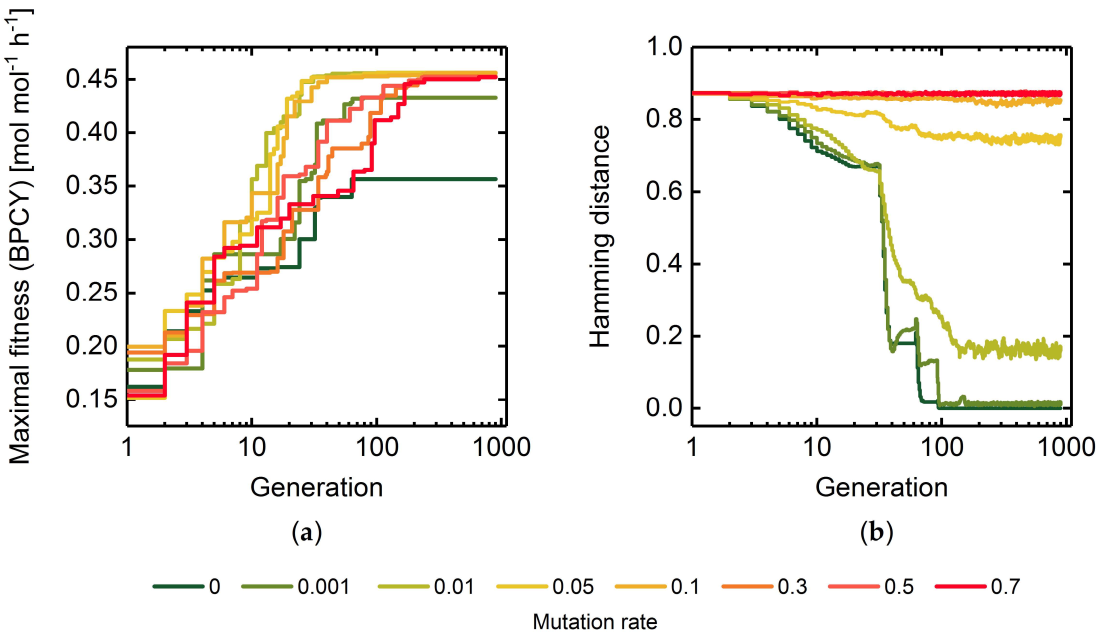

Figure S3: Maximal fitness (**a**) and hamming distance (**b**) across the populations of every thread in each generation using mutation rates between 0 and 0.7. Deletion of maximally five reactions were allowed while using succinate BPCY as the engineering objective. Here, a selection rate of 0.75 was employed.

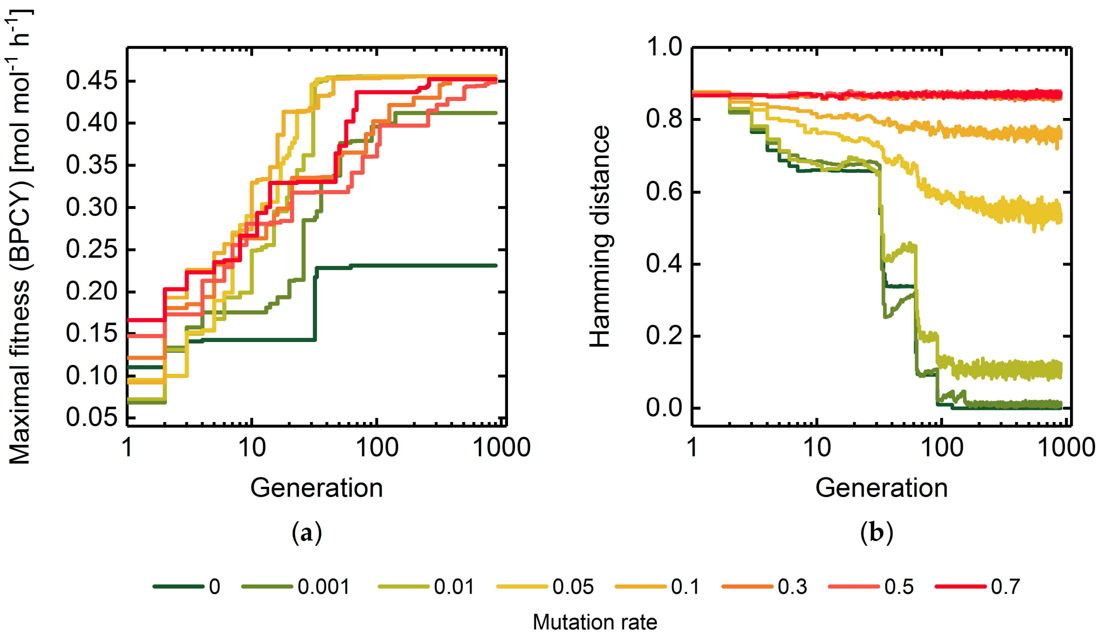

Figure S4: Maximal fitness (**a**) and hamming distance (**b**) across the populations of every thread in each generation using mutation rates between 0 and 0.7. Deletion of maximally five reactions were allowed while using succinate BPCY as the engineering objective. Hamming distance progressions for mutation rates 0.5 and 0.7 overlap each other. Here, a population size of 10 was employed.

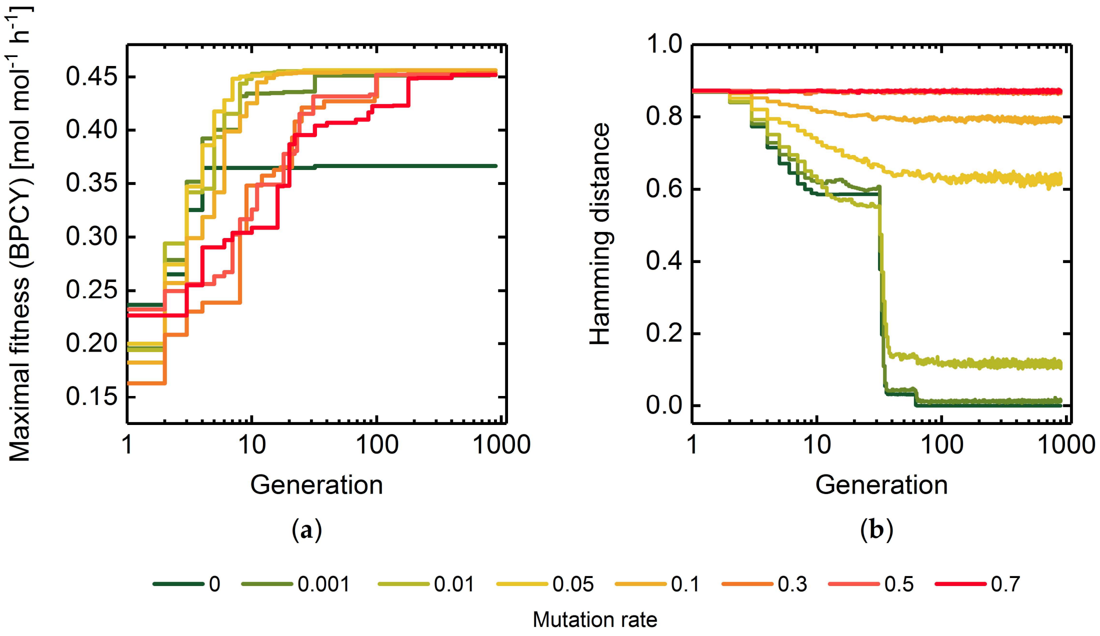

Figure S5: Maximal fitness (**a**) and hamming distance (**b**) across the populations of every thread in each generation using mutation rates between 0 and 0.7. Deletion of maximally five reactions were allowed while using succinate BPCY as the engineering objective. Hamming distance progressions for mutation rates 0.5 and 0.7 overlap each other. Here, a population size of 50 was employed.

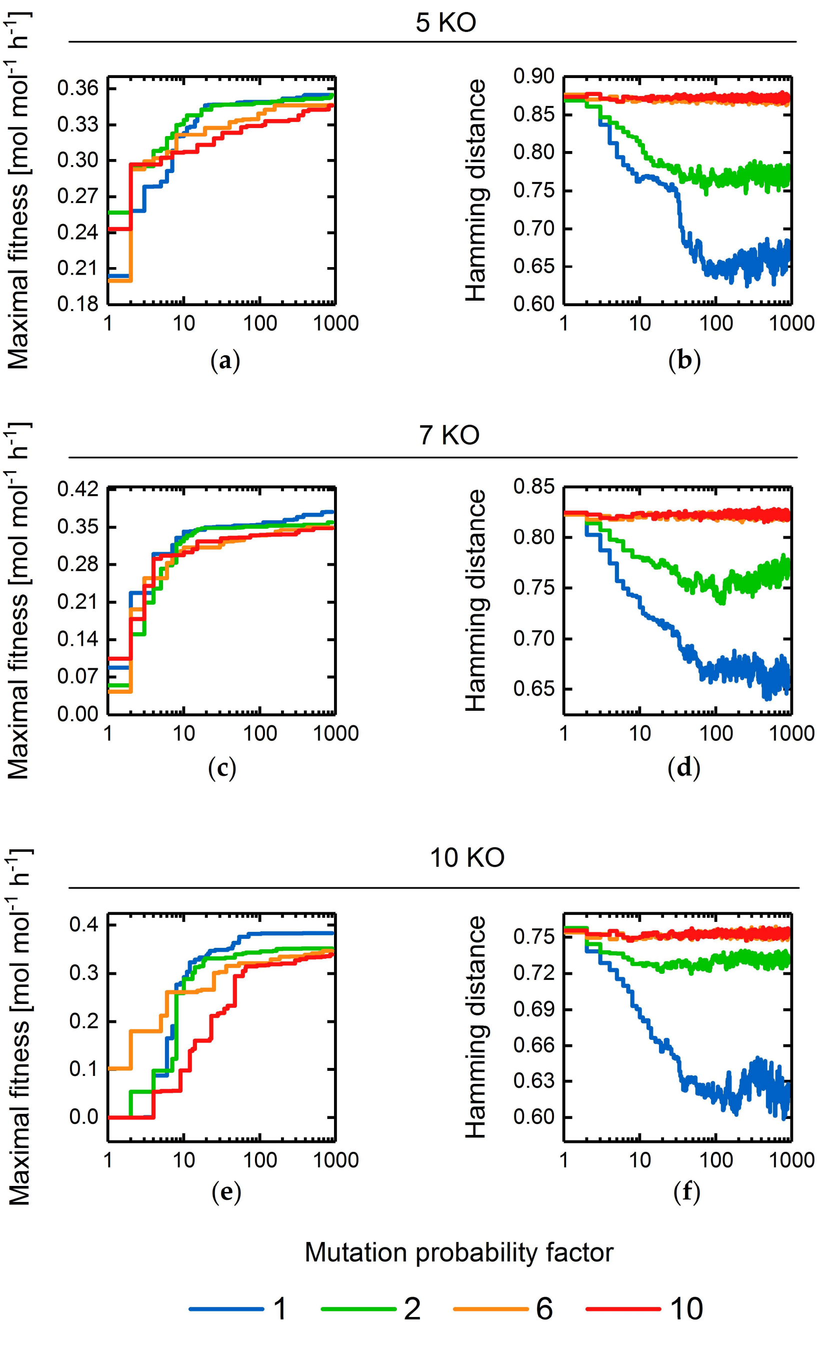

Figure S6: Maximal fitness (BPCY) (**a**, **c**, **e**) and Hamming distance (**b**, **d**, **f**) progressions for GA runs applying five, seven and ten maximal allowable reaction deletions as well as an adaptive mutation probability approach with different ranges between the minimal and maximal mutation rate, all centering around a mutation rate of 0.05. The color code denotes the factor between the minimally and maximally allowable mutation probability. The *E. coli* core model and ethanol BPCY as the engineering objective were employed.

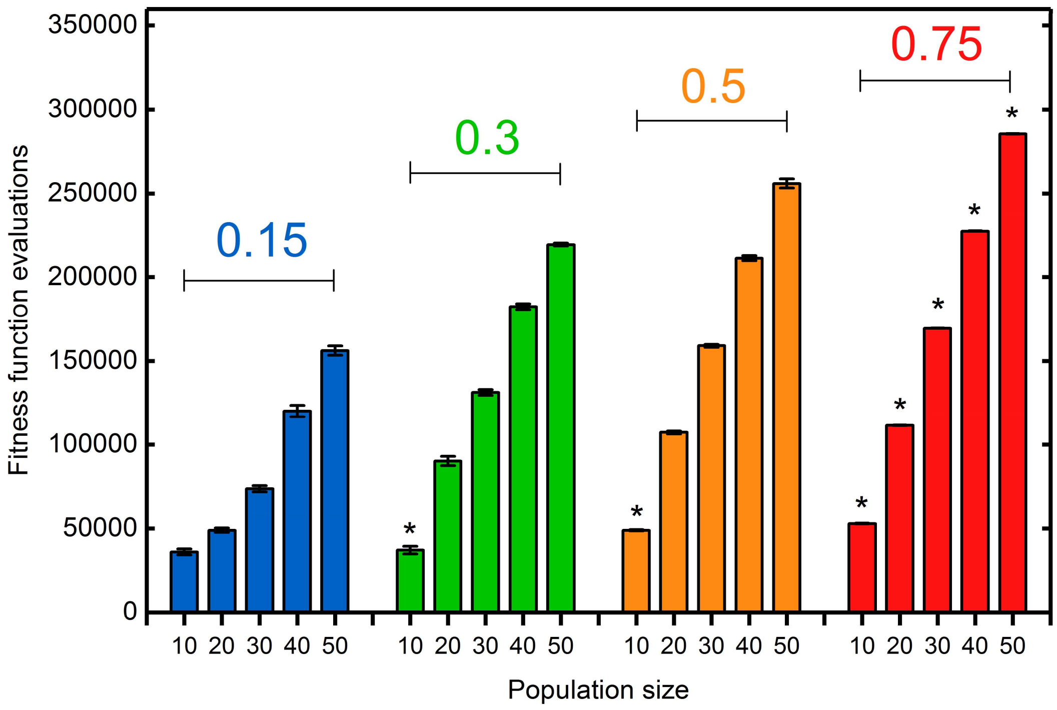

Figure S7: Total number of fitness function evaluations after 900 generations of GA runs applying population sizes between 10 and 50. Bars are clustered according to the employed selection rate (colored number). Error bars show the standard deviation among five replicates for each population size – selection rate pair. Asterisks denote parameter pairs with which the globally maximal fitness of 0.48 $mol\,mol^{-1}\,h^{-1}$ was not reached in every replicate GA run after 900 generations. Succinate BPCY was used as the engineering objective. Intervention set size was seven.

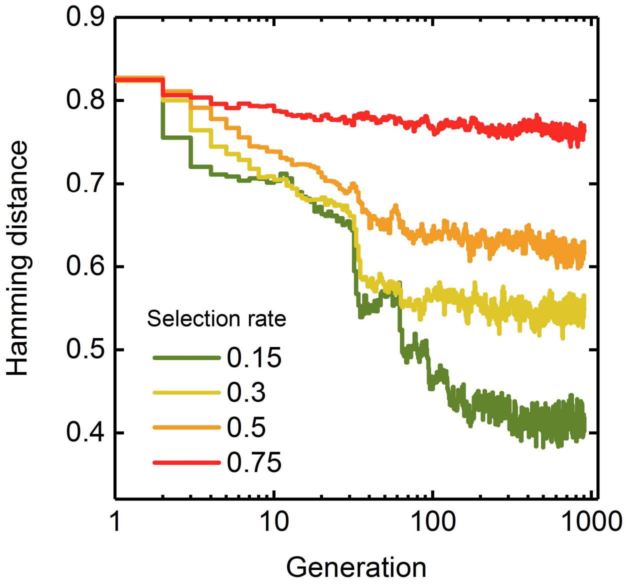

Figure S8: Hamming distance progressions for GA runs using selection rates between 0.15 and 0.75 at a population size of 20. The *E. coli* core model and succinate BPCY as the engineering objective were employed.

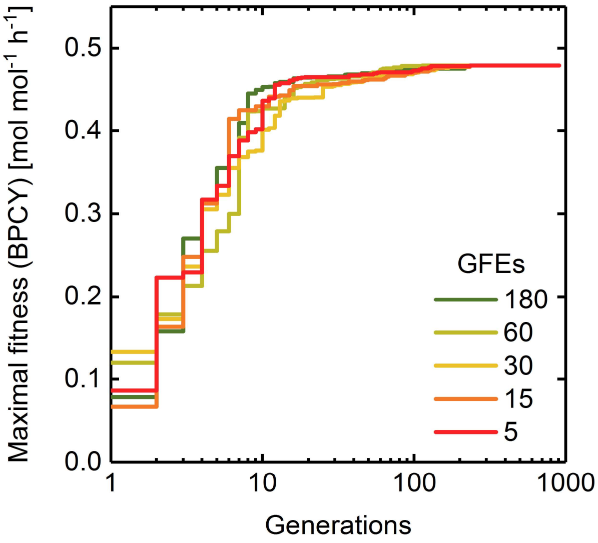

Figure S9: Maximal fitness progressions for GA runs using various numbers of Gene-Flow-Events (GFEs) at a constant total number of 900 generations. The *E. coli* core model and succinate BPCY as the engineering objective were employed.

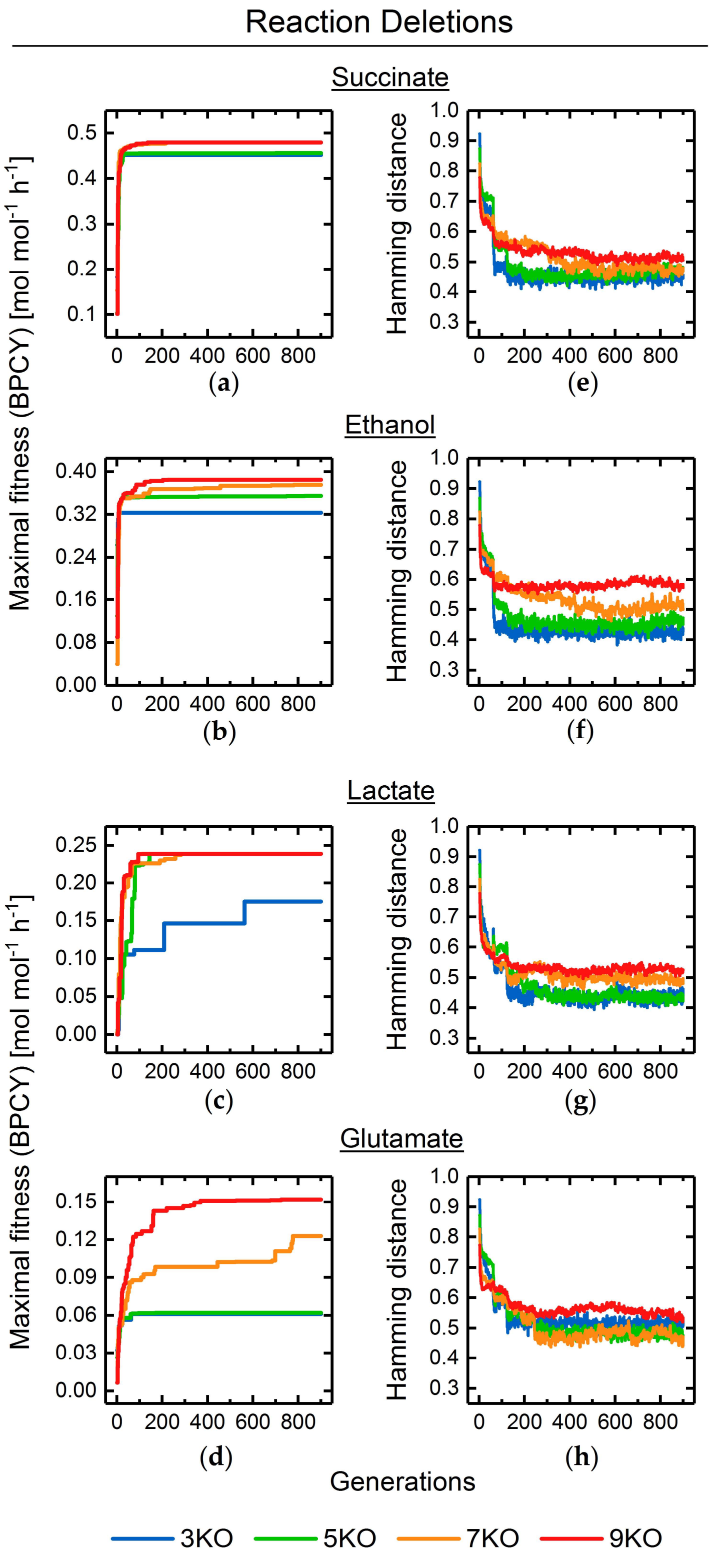

Figure S10: Maximal fitness (BPCY) (**a**, **b**, **c**, **d**) and Hamming distance (**e**, **f**, **g**, **h**) progressions for GA runs applying three, five, seven and nine maximal allowable reaction deletions for optimizing overproduction of succinate, ethanol, lactate and glutamate. The *E. coli* core model and target product BPCY as the engineering objective were employed.

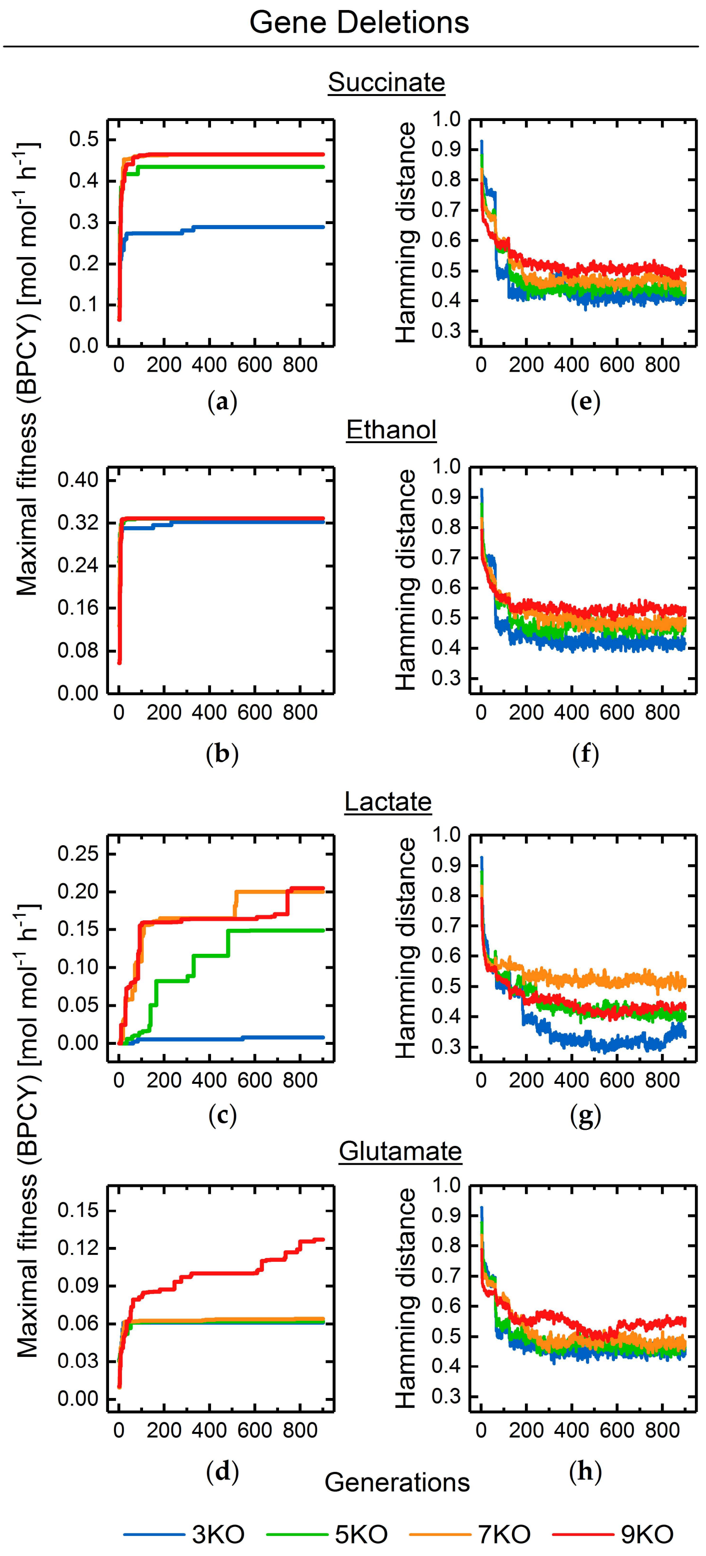

Figure S11: Maximal fitness (BPCY) (**a**, **b**, **c**, **d**) and Hamming distance (**e**, **f**, **g**, **h**) progressions for GA runs applying three, five, seven and nine maximal allowable gene deletions for optimizing overproduction of succinate, ethanol, lactate and glutamate. The *E. coli* core model and target product BPCY as the engineering objective were employed.

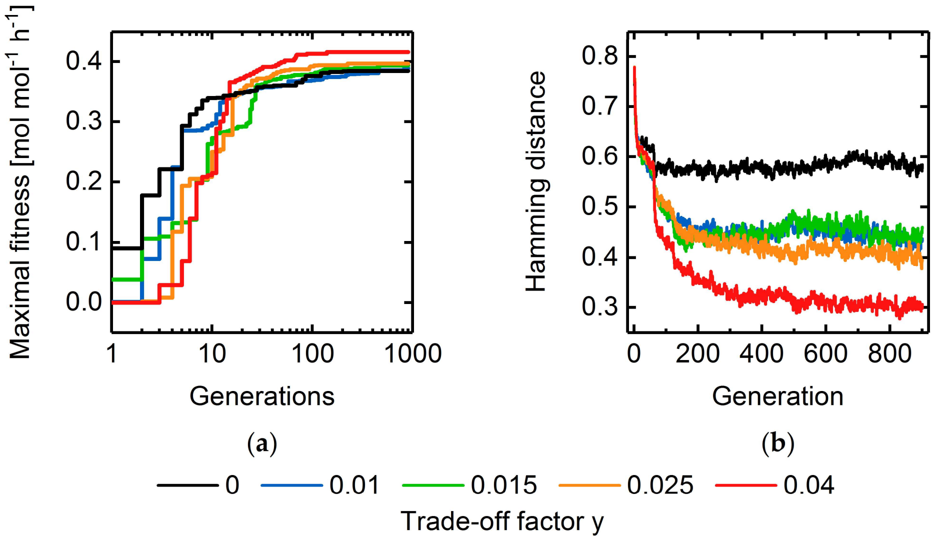

Figure S12: Maximal fitness (**a**) and hamming distance (**b**) progressions for GA runs using trade-off factors of 0, 0.01, 0.015, 0.025 and 0.04 aiming to minimize the final intervention set size. *E. coli* core model and ethanol BPCY as the engineering objective, were employed. The maximally allowable intervention set size was set to nine for each simulation.

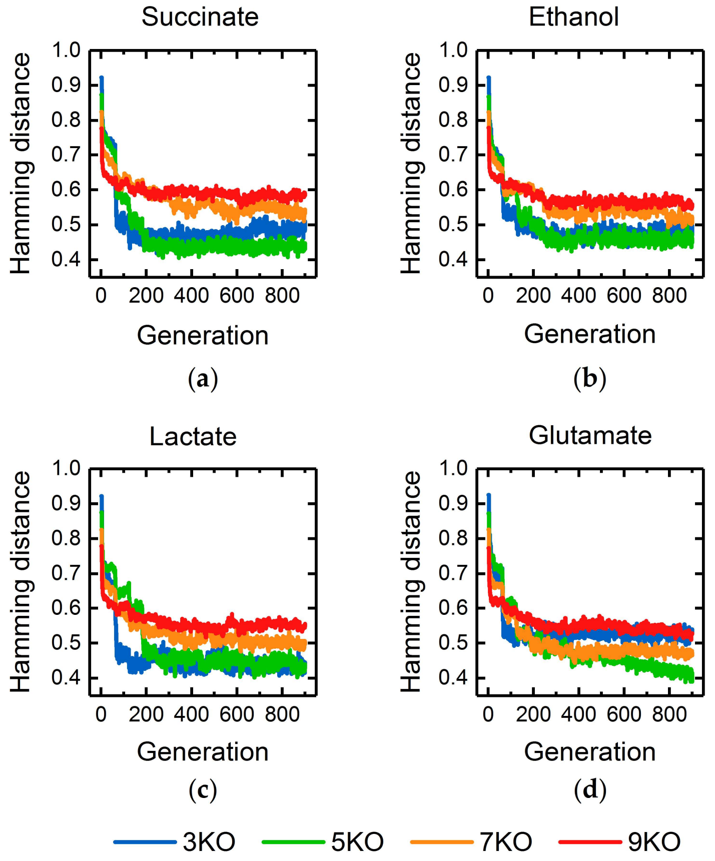

Figure S13: Hamming distance progressions for GA runs applying three, five, seven and nine maximal allowable reaction deletions for optimizing overproduction of succinate (**a**), ethanol (**b**), lactate (**c**) and glutamate (**d**). The *E. coli* core model and multiple objective function approach as the engineering objective, including target product BPCY, growth-coupling strength and target production rate at maximal growth, were employed.

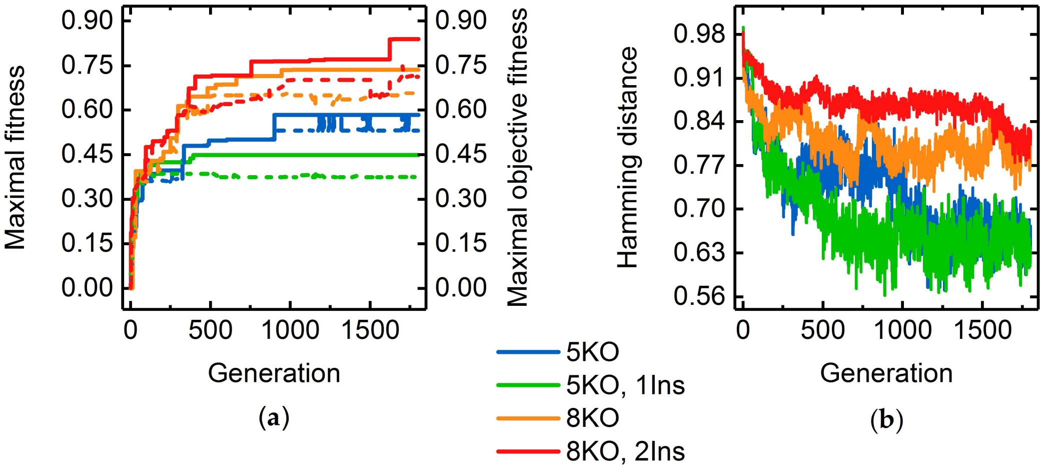

Figure S14: Maximal fitness and objective fitness (**a**) as well as hamming distance (**b**) progressions for GA runs using the *E. coli* GEM *i*JO1366 and different numbers of maximally allowable gene deletions and novel reaction insertions. A multiple objective function approach combining the optimization of succinate BPCY, GCS and succinate production rate at maximal growth was employed. Simultaneously, the intervention set size was minimized using a trade-off *y* of 0.1.

References

1. Long, M. R.; Reed, J. L. Improving flux predictions by integrating data from multiple strains. *Bioinformatics* **2016**, *33*, btw706, doi:10.1093/bioinformatics/btw706.
2. Ishii, N.; Nakahigashi, K.; Baba, T.; Robert, M.; Soga, T.; Kanai, A.; Hirasawa, T.; Naba, M.; Hirai, K.; Hoque, A.; Ho, P. Y.; Kakazu, Y.; Sugawara, K.; Igarashi, S.; Harada, S.; Masuda, T.; Sugiyama, N.; Togashi, T.; Hasegawa, M.; Takai, Y.; Yugi, K.; Arakawa, K.; Iwata, N.; Toya, Y.; Nakayama, Y.; Nishioka, T.; Shimizu, K.; Mori, H.; Tomita, M. Multiple high-throughput analyses monitor the response of *E. coli* to perturbations. *Science (80-.).* **2007**, *316*, 593–597, doi:10.1126/science.1132067.
3. Alter, T. B.; Blank, L. M.; Ebert, B. E. Determination of growth-coupling strategies and their underlying principles. *bioRxiv* **2018**, doi:10.1101/258996.
4. Pharkya, P.; Burgard, A. P.; Maranas, C. D. OptStrain : A computational framework for redesign of microbial production systems. *Genome Res.* **2004**, 2367–2376, doi:10.1101/gr.2872004.
5. Kim, J.; Reed, J. L.; Maravelias, C. T. Large-scale bi-level strain design approaches and mixed-integer programming solution techniques. *PLoS One* **2011**, *6*, doi:10.1371/journal.pone.0024162.
6. Moretti, S.; Martin, O.; Van Du Tran, T.; Bridge, A.; Morgat, A.; Pagni, M. MetaNetX/MNXref - Reconciliation of metabolites and biochemical reactions to bring together genome-scale metabolic networks. *Nucleic Acids Res.* **2016**, *44*, D523–D526, doi:10.1093/nar/gkv1117.
7. Schellenberger, J.; Park, J. O.; Conrad, T. M.; Palsson, B. T. BiGG: A Biochemical Genetic and Genomic knowledgebase of large scale metabolic reconstructions. *BMC Bioinformatics* **2010**, *11*, doi:10.1186/1471-2105-11-213.
8. Kanehisa, M.; Furumichi, M.; Tanabe, M.; Sato, Y.; Morishima, K. KEGG: New perspectives on genomes, pathways, diseases and drugs. *Nucleic Acids Res.* **2017**, *45*, D353–D361, doi:10.1093/nar/gkw1092.
9. Flamholz, A.; Noor, E.; Bar-Even, A.; Milo, R. EQuilibrator - The biochemical thermodynamics calculator. *Nucleic Acids Res.* **2012**, *40*, 770–775, doi:10.1093/nar/gkr874.

© 2018 by the authors. Licensee MDPI, Basel, Switzerland. This article is an open access article distributed under the terms and conditions of the Creative Commons Attribution (CC BY) license (http://creativecommons.org/licenses/by/4.0/).
